# Mechanotransduction through LFA-1 controls upstream migration in the KG-1a stem cell line

**DOI:** 10.1242/jcs.265036

**Published:** 2026-10-14

**Authors:** Ai Mochida, Emily Valerio, Janis K. Burkhardt, Daniel A. Hammer

**Affiliations:** ^1^ Department of Bioengineering, University of Pennsylvania, Philadelphia, PA 19104, USA; ^2^ Department of Chemical and Biomolecular Engineering, University of Pennsylvania, Philadelphia, PA 19104, USA; ^3^ Department of Pathology and Laboratory Medicine, Children's Hospital of Philadelphia and Perelman School of Medicine, University of Pennsylvania, Philadelphia, PA 19104, USA

**Keywords:** Migration, Integrins, Leukocyte, Shear flow, Mechanotransduction, Actin

## Abstract

Immune cell recruitment into tissues requires cells to migrate on the endothelium while overcoming shear forces. Many extravasating cells, including T lymphocytes and hematopoietic stem cells, migrate upstream against the direction of shear flow on ICAM-1. Upstream migration has been shown to depend on intracellular signaling through Crk family adaptor proteins following engagement of lymphocyte function-associated antigen-1 (LFA-1). Because upstream migration is an important illustration of leukocyte mechanotransduction, we sought to identify key intracellular regulators of upstream migration using CRISPR/Cas9 gene editing and the KG-1a stem cell line. We made targeted deletions of molecules that function downstream of LFA-1 to regulate actin polymerization, link integrin signaling to the actin cytoskeleton, or form scaffolding complexes with Crk proteins. We found that CrkL, CasL (also known as HEF1 or NEDD9), L-plastin (LCP1) and RIAM (APBB1IP) are crucial for dynamic F-actin-rich lamellipodia and upstream migration. Although WASP (WAS), Hem1 (NCKAP1L), EVL and vinculin were found to be dispensable for upstream migration, pan-formin inhibition with SMIFH2 revealed a role for formins in upstream migration. This study illustrates the role of LFA-1 mechanotransduction in driving F-actin assembly at the leading edge to drive upstream migration.

## Teaser

**Summary:** Many extravasating immune cells can migrate upstream, or against the direction of flow, on the endothelium. Using CRISPR/Cas9, we identify mechanotransducers that are essential for upstream migration.

## INTRODUCTION

Immune cells frequently transduce mechanical forces from their environment into biochemical signals to guide decisions such as cell adhesion, migration, differentiation, metabolism and proliferation. At the same time, cells also generate forces through motor proteins that regulate cell morphology, traction and the assembly of the actin cytoskeleton. Mechanotransduction is a dynamic process coupling external mechanical forces with cell signaling, the actin cytoskeleton and cell-generated internal forces (Chen, 2008). One important example of mechanotransduction is migration on the apical surface of the endothelium, where leukocytes use activated integrins that sense shear stress from blood flow to guide their motility, ultimately leading to extravasation.

Leukocytes exit blood vessels and enter tissues to defend against pathogens and perform immunosurveillance. This process requires a multi-step adhesion cascade involving selectin-mediated rolling, integrin-mediated adhesion, migration on the apical surface of endothelium and transmigration (Ley et al., 2007; Schenkel et al., 2004). The apical surface of the endothelium displays the adhesion molecules intercellular adhesion molecule-1 (ICAM-1) and vascular cell adhesion molecule-1 (VCAM-1), which bind to the leukocyte integrins lymphocyte function-associated antigen-1 (LFA-1) and very late antigen-4 (VLA-4), respectively. In landmark studies, it has been shown that human primary CD4^+^ T lymphocytes can preferentially migrate upstream – against the direction of shear flow – on surfaces coated with ICAM-1 (Valignat et al., 2014, 2013). In contrast, CD4^+^ T lymphocytes have been found to preferentially migrate downstream (with the direction of shear flow) on VCAM-1 surfaces (Dominguez et al., 2015; Hornung et al., 2020; Steiner et al., 2010). The integrin-dependent regulatory cascades that control these distinct directional responses are largely unknown.

The ability to migrate upstream appears to be preserved across many leukocyte lineages (Buffone et al., 2023). In addition to human primary CD4^+^ T lymphocytes, upstream migration has been observed in many cell types that migrate from blood vessels to tissues, including murine primary CD4^+^ T lymphocytes, hematopoietic stem and progenitor cells (HSPCs), and the KG-1a cell line, an immortalized human HSPC-like cell line with an integrin receptor profile similar to that of T lymphocytes (Buffone et al., 2018; Roy et al., 2020). When Mac-1 ligand binding is blocked, polymorphonuclear neutrophils (PMNs) and the HL-60 neutrophil-like cell line are also able to migrate upstream (Buffone et al., 2019). Upstream migration has been further observed to occur with primary marginal zone B lymphocytes in the spleen as they migrate towards the follicle (Tedford et al., 2017). Therefore, all extravasating cells expressing the integrin LFA-1 share the ability to overcome shear forces and migrate upstream. Numerous examples of upstream migration have been observed *in vivo* (Bartholomaus et al., 2009; Phillipson et al., 2009; Smyth et al., 2024). Upstream migration appears to provide leukocytes a means of efficient tissue surveillance (Abadier et al., 2015; Barreiro et al., 2021) and perhaps facilitates faster rates of transendothelial migration (Anderson et al., 2019).

Previous work supports a model of upstream migration in which signaling cascades are activated following LFA-1 engagement to ICAM-1-bearing surfaces. According to this mode, shear flow exerts forces through LFA-1–ICAM-1 bonds, leading to actin polymerization and upstream migration. Upstream migration in murine CD4^+^ T lymphocytes requires the CT10 regulator of kinase (Crk) family adaptor proteins, particularly the CrkL isoform, and the E3 ubiquitin ligase scaffold protein c-Cbl, but not phosphoinositide 3-kinase (PI3K) (Roy et al., 2020). Deletion of Crk proteins perturbs ICAM-1-induced cell spreading and F-actin polymerization at the lamellipod (Roy et al., 2020, 2018). Surprisingly, inhibition of Arp2/3-dependent branched actin networks does not affect the ability of human CD4^+^ T lymphocytes to orient and migrate against the direction of shear flow on ICAM-1 (Kim and Hammer, 2019). However, the biochemical signaling pathway downstream of LFA-1 that leads to upstream migration is not completely known.

Large-scale CRISPR screens in primary T cells and HL-60 cells have previously identified drivers of adhesion and context-dependent motility (Belliveau et al., 2023; Johansen et al., 2022). In this study, we performed targeted CRISPR/Cas9 deletions in KG-1a cells to investigate other LFA-1 intermediates involved in upstream migration. We sought to answer two questions through a targeted screen. First, in addition to Crk family proteins, are there other molecules downstream of LFA-1 that physically or biochemically link LFA-1 to actuating networks that play a key role in regulating upstream migration? Second, can we probe the contributions of various actin polymerization effectors, including the Arp2/3 complex, Ena/VASP proteins and formins, in upstream migration? Thus, we targeted molecules in two broad families: LFA-1 mechanotransducers and actin regulators. The LFA-1 mechanotransducers we selected for analysis included molecules interacting with Crk family or c-Cbl proteins such as CasL (also known as HEF1 or NEDD9) and molecules linking integrins to the actin cytoskeleton such as vinculin (VCL), actin-bundling protein L-plastin (LCP1) and Rap1-interacting molecule (RIAM, also known as APBB1IP). We also investigated mediators of actin polymerization including nucleation-promoting factors (NPFs) in the Wiskott–Aldrich syndrome protein (WASP, also known as WAS) and SCAR/WAVE families, as well as elongation promoting factors in the Ena/VASP family. In each case, candidate proteins were deleted from KG-1a cells, and cell migration and F-actin dynamics at the leading edge were analyzed under shear flow, with the goal of understanding how molecules involved in integrin mechanotransduction and actin polymerization modulate upstream migration. We found that CrkL, CasL, L-plastin and RIAM were crucial for upstream migration, but surprisingly, regulators of actin polymerization were not. By combining tools for molecular manipulation with measurements of motility, our methodology provides insight into the mechanisms by which immune cells sense and respond to external forces.

## RESULTS

The signaling pathways emanating from LFA-1 engagement that control upstream migration are not fully understood. Using CRISPR/Cas9 gene editing to target candidate molecules, we generated a panel of knockout (KO) KG-1a cell lines and tested the ability of these cells to migrate under shear flow conditions. Our panel included a negative control line generated using a non-targeting (NT) guide RNA (gRNA). Comparison of these control NT cells with unedited wild-type (WT) cells showed that the methods used for gene editing did not affect cell migration or alter integrin expression levels between WT and NT cells (Fig. S1).

### L-plastin, CasL and RIAM reverse the direction of cell migration under shear flow

Because the Crk family adaptor protein CrkL has previously been shown to mediate upstream migration (Roy et al., 2020), we used CrkL-deleted KG-1a cells as a positive control. In addition, we focused on binding partners for CrkL, as well as other molecules that transmit signals from LFA-1, including L-plastin, CasL, RIAM and vinculin. KG-1a KO cells lacking these proteins were generated using CRISPR/Cas9 gene editing. Deletion was confirmed by genomic DNA sequencing and immunoblotting (Fig. 1A). Importantly, these deletions had little to no effect on cell surface expression levels of the integrins LFA-1 or VLA-4 (Fig. S2A,B). Random migration speed, the motility coefficient and persistence time in the absence of shear flow was measured on ICAM-1 surfaces (Fig. 1B–E). Deleting L-plastin decreased migration speed and increased persistence time compared to NT cells but did not affect the random motility coefficient. In contrast, deleting CrkL, CasL, vinculin or RIAM did not affect migration speed, the random motility coefficient or persistence time on ICAM-1 (Fig. 1C–E). Thus, aside from L-plastin, deletions in these LFA-1 mechanotransducing molecules did not affect the dynamics of random cell motility.

**Fig. 1.**
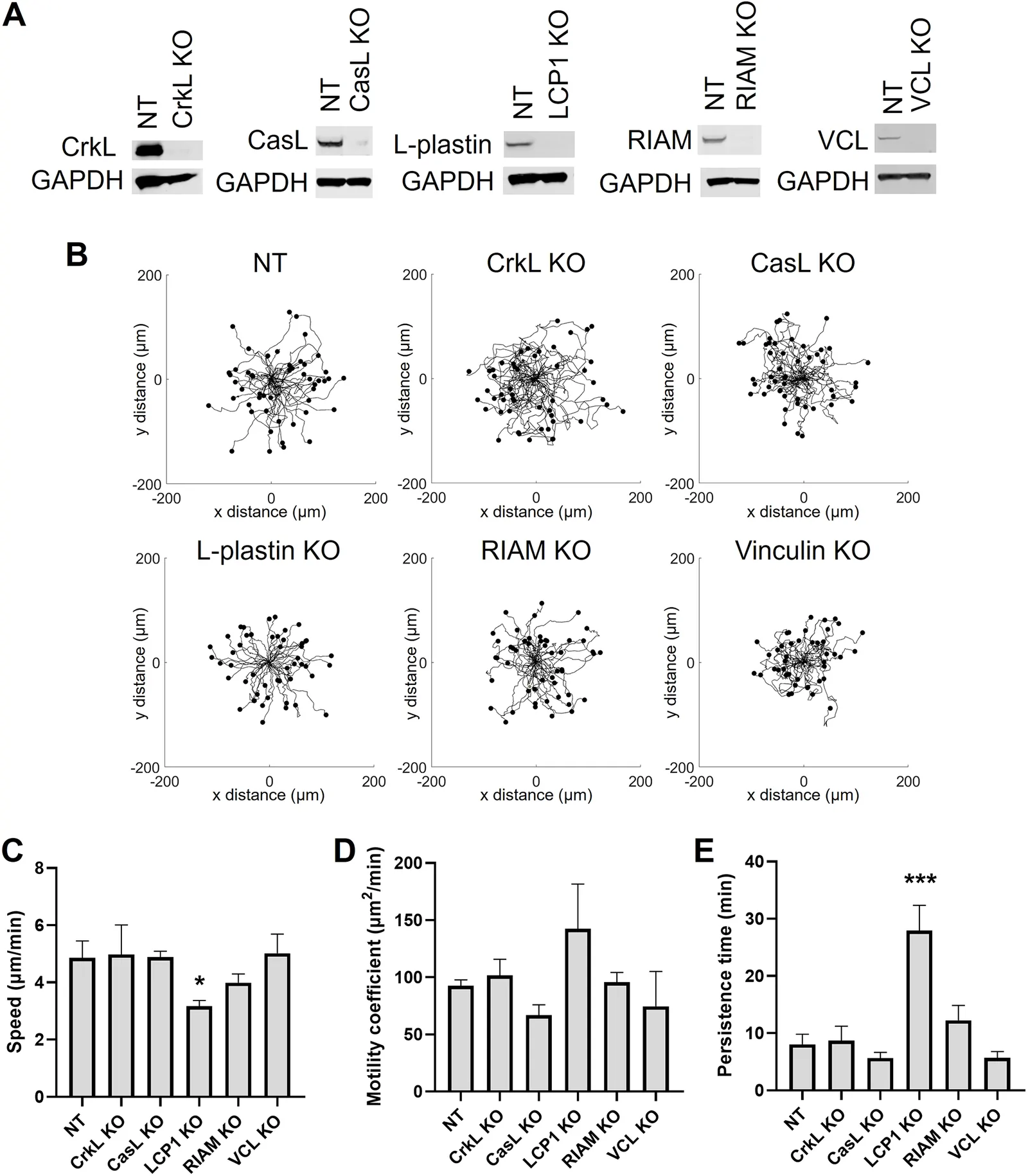
**Deleting CrkL, CasL, RIAM or vinculin does not affect random migration on ICAM-1.** (A) CrkL, CasL, L-plastin (LCP1), RIAM and vinculin (VCL) were deleted in KG-1a cells, and deletion was confirmed by western blotting. GAPDH was used as a loading control. Blots shown are representative of three independent experiments. (B) Random migration on ICAM-1 surfaces was assessed for the indicated cell lines, and representative cell tracks are shown. Dots indicate track end points. (C–E) Population-level parameters (C) speed, (D) motility coefficient and (E) persistence time were calculated from tracks as in B. Bars represent mean±s.d. using three independent experiments with at least 30 cells analyzed per experiment. Statistics relative to NT control were calculated using a one-way ANOVA with Dunnett's multiple comparisons (*P<0.05; ***P<0.001).

We then characterized migration of KG-1a cells lacking LFA-1 mechanotransducers under shear flow to measure upstream cell fraction, the mean migration index and directionality (Fig. 2). The mean migration index quantified the degree of upstream or downstream migration, whereas directionality quantified the degree of migration in a straight path. As anticipated, NT control KG-1a cells preferentially migrated upstream. Deletion of CrkL disrupted this behavior, consistent with previous findings in murine CD4^+^ T lymphocytes (Roy et al., 2020). Thus, our results on KG-1a directional migration recapitulate work performed in murine CD4^+^ T lymphocytes and support a conserved mechanism for upstream migration in extravasating immune cells. We also found CasL, a binding partner for CrkL, to be essential for upstream migration in KG-1a cells. In addition to CrkL and CasL, we observed that KG-1a cells lacking L-plastin or RIAM, which link LFA-1 to the actin cytoskeleton, failed to migrate upstream, as indicated by the decreased upstream fractions and positive mean migration indices, compared to NT cells (Fig. 2). In contrast, vinculin was not required for upstream migration, perhaps suggesting a diverse role in cell organization beyond engaging LFA-1. Cell tracks indicated that CasL KO and L-plastin KO cells migrated in a more directional manner than NT cells, whereas vinculin KO cells tended to make more turns (Fig. 2C). Shear flow experiments performed at 400 s^−1^ also showed consistent migratory phenotypes on ICAM-1 (Fig. S3). Using independently derived clones, we consistently observed that deleting CrkL, CasL, L-plastin or RIAM, but not vinculin, reversed upstream migration on ICAM-1 at a shear flow rate of 800 s^−1^ (Fig. S4).

**Fig. 2.**
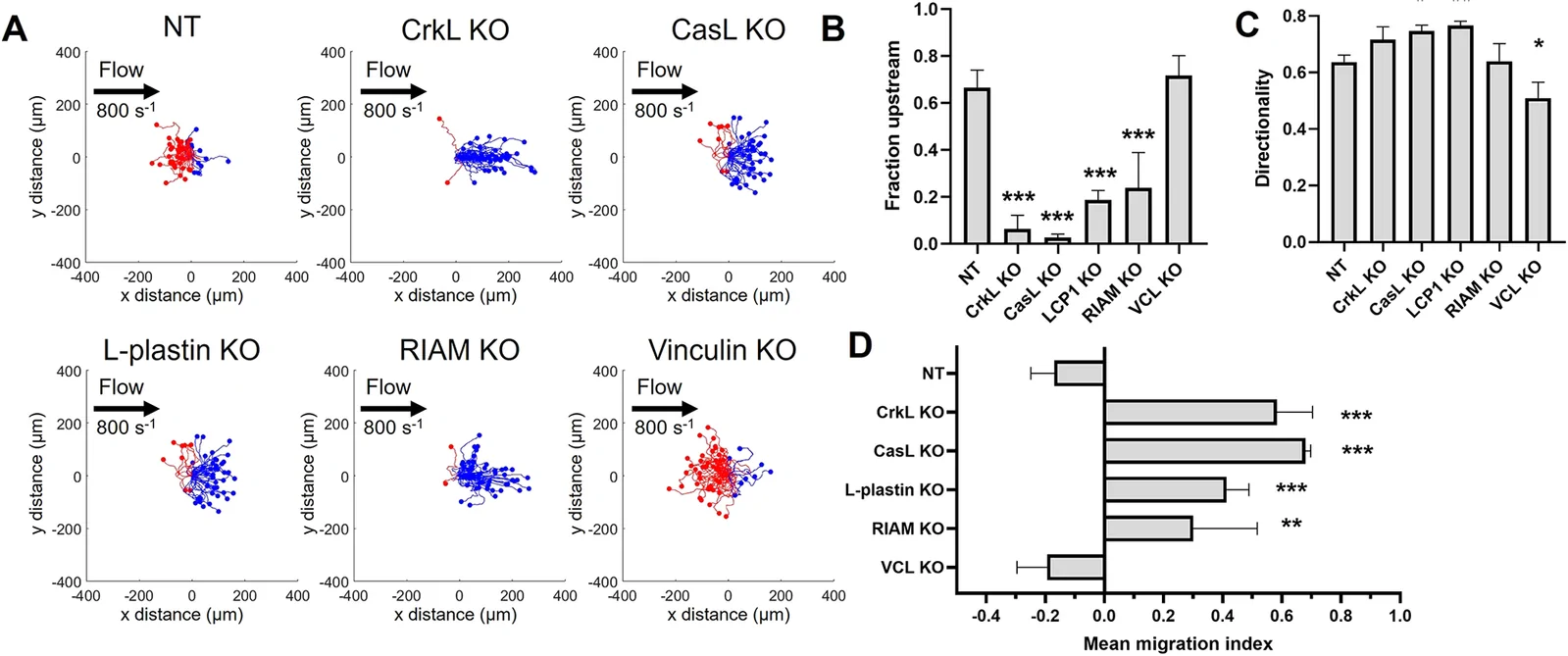
**CrkL, CasL, L-plastin and RIAM KO reverses KG-1a upstream migration on ICAM-1.** (A) Representative plots of KG-1a NT, CrkL KO, CasL KO, L-plastin (LCP1) KO, vinculin (VCL) KO and RIAM KO cells migrating on ICAM-1 surfaces under 800 s^−1^ shear flow (left to right). Red tracks indicate cells migrating upstream against the direction of flow, while blue tracks indicate cells migrating downstream with the direction of flow. Dots indicate track end points. (B–D) Migration characteristics were quantified from tracks as in A for each cell line: (B) fraction of cells migrating upstream, (C) mean directionality and (D) mean migration index. Bars represent mean±s.d. using three independent experiments with at least 30 cells analyzed per experiment. Statistics relative to NT control were calculated using a one-way ANOVA with Dunnett's multiple comparisons (*P<0.05; **P<0.01; ***P<0.001).

### Formins, but not Arp2/3 or Ena/VASP family actin regulatory molecules, mediate upstream migration

The roles of actin NPFs in the WASP and SCAR/WAVE protein families and the linear actin filament elongation factors in the Ena/VASP protein family in cell migration have been previously explored under static conditions but not under shear flow (Bear et al., 2000; Belliveau et al., 2023; Billadeau and Burkhardt, 2006; Brunetti et al., 2022; Damiano-Guercio et al., 2020; Estin et al., 2017; Graziano et al., 2019). We therefore generated KG-1a cells lacking actin regulatory molecules WASP, Hem1 (also known as NCKAP1L) or EVL. Deletion of the targeted molecules was confirmed by genomic DNA sequencing and immunoblotting (Fig. 3A). Deletion of Hem1 is known to degrade other subunits of the WAVE complex, including WAVE2 (WASF2), which is the WAVE complex protein predominantly expressed in hematopoietic cells (Graziano et al., 2019). In keeping with this, we found that Hem1 KO KG-1a cells expressed very low levels of WAVE2 (Fig. 3B). KO lines had normal expression levels of integrins relevant for cell adhesion (Fig. S2C,D).

**Fig. 3.**
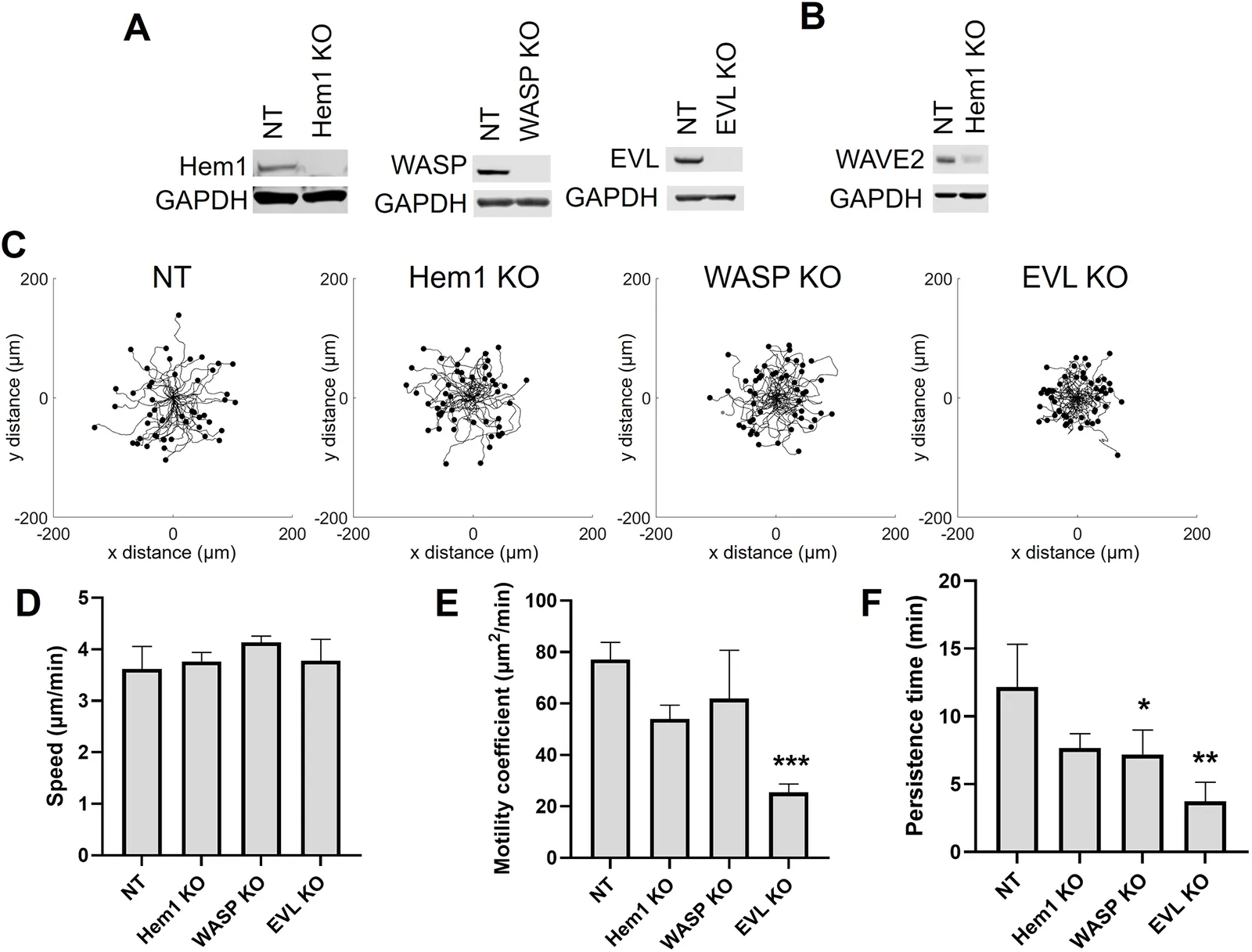
**Deleting WASP and EVL affects random migration on ICAM-1.** (A) WASP, Hem1 and EVL were deleted in KG-1a cells, and deletion was confirmed by western blotting. GAPDH was used as a loading control. (B) Hem1 KO cell lysates were also immunoblotted for WAVE2. Blots shown in A and B are representative of three independent experiments. (C) Random migration on ICAM-1 surfaces was assessed for the indicated cell lines, and representative cell tracks are shown. Dots indicate track end points. (D–F) Population-level parameters (D) speed, (E) motility coefficient and (F) persistence time were calculated from tracks as in C. Bars represent mean±s.d. using three independent experiments with at least 30 cells analyzed per experiment. Statistics relative to NT control were calculated using a one-way ANOVA with Dunnett's multiple comparisons (*P<0.05; **P<0.01; ***P<0.001).

Random migration of KG-1a WASP KO, Hem1 KO and EVL KO cells was characterized on ICAM-1 surfaces (Fig. 3C). Migration speed was unaffected by deletion of WASP, Hem1 or EVL, but the random motility coefficient was diminished moderately in Hem1 KO cells and strongly in EVL KO cells (Fig. 3D,E). Persistence time was strongly decreased in EVL KO cells and moderately reduced in Hem1 KO and WASP KO cells, though only the effects on EVL KO and WASP KO cells reached statistical significance (Fig. 3F). Using independently derived clones, we consistently observed that deleting EVL or WASP affected random migration (Fig. S5). In contrast to the differences observed in random migration, deletions of the actin regulatory molecules in our CRISPR/Cas9 KO panel did not affect upstream migration or directionality on ICAM-1 under shear flow compared to NT cells (Fig. 4A–D). Interestingly, these data show that key molecules contributing to polymerizing branched and linear actin filaments are dispensable for upstream migration on ICAM-1.

**Fig. 4.**
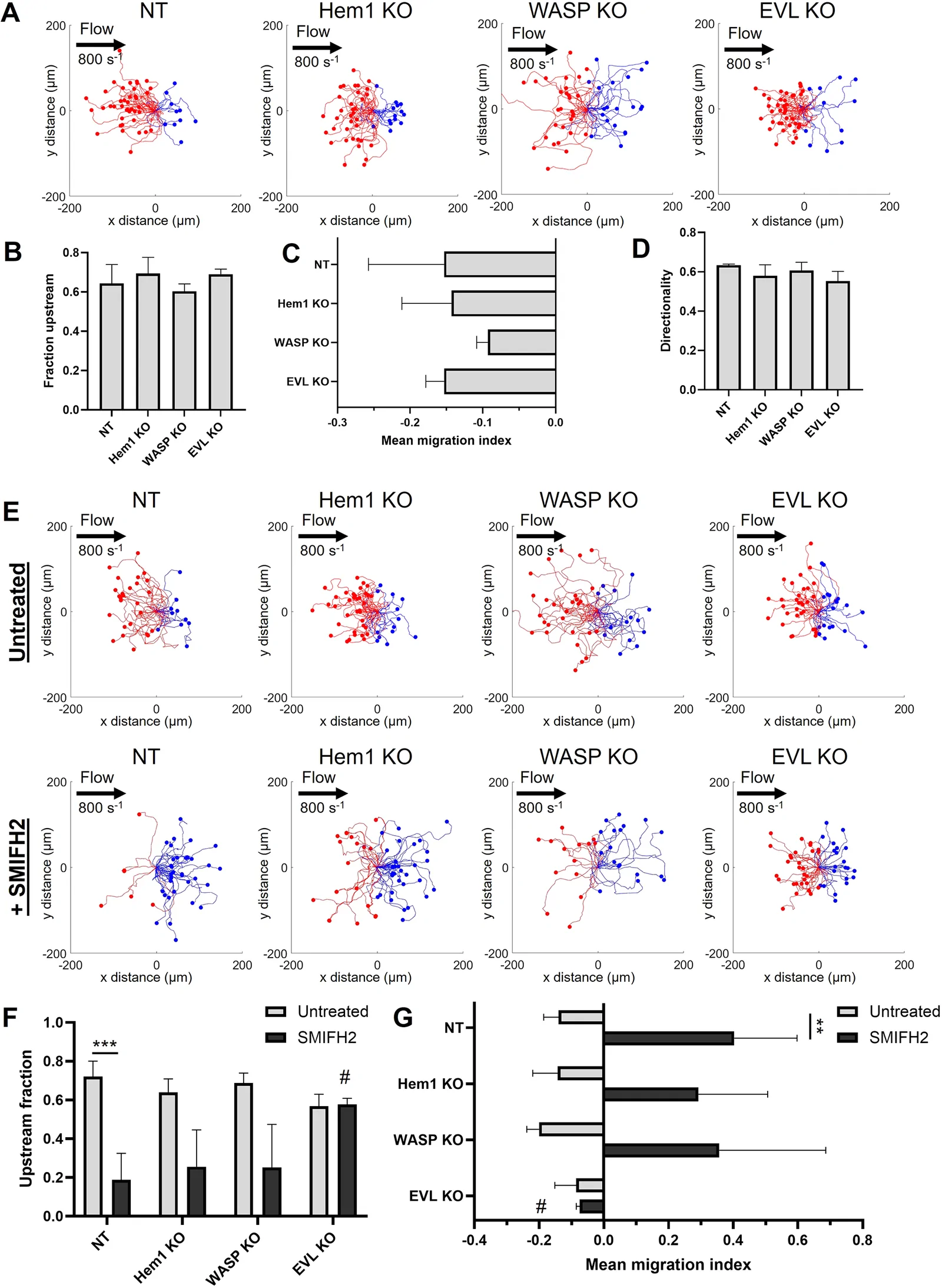
**Deleting WASP, EVL and Hem1 does not affect KG-1a migration under shear flow.** (A) Representative plots of NT, Hem1 KO, WASP KO and EVL KO cells migrating on ICAM-1 surfaces under 800 s^−1^ shear flow (left to right). (B–D) Migration characteristics were quantified from tracks as in A for each cell line: (B) fraction of cells migrating upstream, (C) mean migration index and (D) mean directionality. (E) Representative plots of NT, Hem1 KO, WASP KO and EVL KO cells migrating on ICAM-1 under 800 s^−1^ shear flow (left to right) following 30 min pre-treatment with DMSO (control, untreated) or 5 μM SMIFH2. Treatment was maintained throughout the experiment. (F) Fraction of cells migrating upstream and (G) mean migration index calculated from tracks as in E are displayed for DMSO (untreated) and 5 μM SMIFH2 conditions. In A and E, red tracks indicate cells migrating upstream against the direction of flow, while blue tracks indicate cells migrating downstream with the direction of flow. Dots indicate track end points. In B–D,F,G, bars represent mean±s.d. using three independent experiments with at least 30 cells analyzed per experiment. In B–D, statistics relative to NT control were calculated using a one-way ANOVA with Dunnett's multiple comparisons. In F,G, statistics between untreated and treated groups were calculated using an unpaired, two-tailed *t*-test (**P<0.01; ***P<0.001), and statistics were calculated using a one-way ANOVA with Dunnett's multiple comparisons relative to NT control within untreated or treated groups (^#^*P*<0.05).

Since deleting actin regulatory molecules within the Arp2/3 complex or Ena/VASP family did not affect upstream migration, we next decided to study formins, another effector of actin polymerization. Formins mediate polymerization of linear actin filaments and can be studied with the pan-formin inhibitor SMIFH2. By treating KO cells in the actin regulatory group with SMIFH2, we aimed to study the dynamics of actin polymerization effectors in mediating migration under shear flow. NT cells treated with 5 μM SMIFH2 migrated primarily downstream compared to untreated NT cells, with a decreased upstream fraction and increased mean migration index (Fig. 4E–G). These results suggest the polymerization of linear actin filaments by formins is important for upstream migration. Next, when WASP KO or Hem1 KO cells were treated with SMIFH2, there were no changes in the upstream fraction or mean migration index compared to SMIFH2-treated NT cells (Fig. 4E–G). In other words, deleting WASP or Hem1 in addition to SMIFH2 treatment did not lead to more downstream migration. However, SMIFH2-treated EVL KO cells retained the ability to migrate upstream (Fig. 4E–G). These results reinforce the compensatory mechanism between Arp2/3 and Ena/VASP, where actin nucleation and elongation are balanced (Rotty and Bear, 2014). EVL deletion may have reduced activity of Ena/VASP, leading to an increase in branched actin nucleation and a dense actin dendritic network. Thus, we propose that SMIFH2-treated EVL KO cells may have generated enough forces at the leading edge to migrate upstream. SMIFH2-treated NT cells with balanced nucleation and elongation, or SMIFH2-treated WASP KO or SMIFH2-treated Hem1 KO cells with reduced nucleation, may not have been able to compensate for the inhibition of formins, leading to downstream migration.

### Distinct morphologies between KO cells migrating upstream and downstream

Table 1 summarizes the effects of deleting LFA-1 mechanotransducers and actin regulatory molecules on KG-1a random migration and upstream migration. Upstream migration on ICAM-1 required CrkL, L-plastin, CasL and RIAM. To understand how cytoarchitecture controls the migratory response under shear flow, we obtained images of cells at a higher level of magnification (Fig. S6A,B, Movie 1). Despite cell variability, we found that KO cells that migrated upstream displayed leading edges containing finger-like protrusions extending from broadly spread lamellipodia, as well as bulbous uropods, similar to the morphology of CD4^+^ T lymphocytes migrating on ICAM-1 (Roy et al., 2020; Valignat et al., 2013). In contrast, KO cells that migrated downstream often displayed leading edges funneled in the direction of shear flow (without finger-like protrusions) and small uropods similar to the morphology of CD4^+^ T lymphocytes migrating on VCAM-1 (Valignat et al., 2014).

**Table 1.** Effects of deletions on random and upstream migration on ICAM-1 compared to control

Group	Knockout	Effects on random migration	Direction of migration under shear flow
LFA-1 mechanotransducers	CrkL	No	Downstream
	CasL	No	Downstream
	L-plastin	Yes	Downstream
	RIAM	No	Downstream
	Vinculin	No	Upstream
Actin regulatory molecules	Hem1	No	Upstream
	WASP	Yes	Upstream
	EVL	Yes	Upstream

While imaging at a higher level of magnification, we observed that KG-1a KO cells displayed blebbing behavior. Bleb-based protrusions are membrane bulges generated via actomyosin contractility and hydrostatic pressure instead of actin polymerization (De Belly and Weiner, 2024; Schick and Raz, 2022). We quantified the percentage of blebbing cells under static and shear flow conditions (Fig. S6C). When comparing the blebbing behavior of NT cells under static and shear flow conditions, we found that shear flow decreased the extent of blebbing. Under static conditions, deleting LFA-1 mechanotransducers or actin regulatory molecules did not significantly alter the extent of blebbing compared to that seen for NT cells. However, under shear flow, we found that Hem1 KO and EVL KO cells displayed higher extent of blebbing compared to NT cells. When comparing each KO under static and shear flow conditions, shear flow strongly increased the extent of blebbing in EVL KO cells and moderately increased the extent of blebbing in Hem1 KO and WASP KO cells. However, there was a high degree of variability, and statistical significance was only reached in EVL KO cells. Taking the blebbing data together, a higher incidence of blebbing was observed in KG-1a cells under shear flow when actin regulatory molecules were deleted. This is likely because diminished actin polymerization leads to reduced cortical tension and increased plasma membrane detachment from the actin cortex under shear flow conditions (Ikenouchi and Aoki, 2022; Obeidy et al., 2020). Nonetheless, we found no correlation between the ability to migrate upstream under shear flow and the tendency to bleb while exposed to shear flow.

### KO cells migrating downstream retract less at the leading edge

To observe F-actin behavior during migration, we transduced our panel of KO cell lines with Lifeact–GFP and imaged the dynamics of actin for ∼3 min as the cells migrated on ICAM-1 under shear flow conditions (Movies 2, 3). Kymographs were generated to analyze the dynamics of the leading edge by drawing a line starting from the centroid of each cell extending in the direction of migration. For the KO cell lines, only those cells migrating in the direction characteristic for the respective cell line (e.g. upstream for WASP KO) were analyzed. NT cells migrating upstream and downstream were analyzed to serve as a control. Upstream-migrating NT cells and KOs displayed F-actin enrichment at the leading edge (Fig. 5A), whereas downstream-migrating NT cells and KOs displayed F-actin enrichment at the uropod and minimal F-actin at the leading edge (Fig. 5B). Kymographs showed that upstream-migrating NT cells and KOs migrated in short bursts, with their leading edge lamellipodia undergoing frequent cycling between protrusions and retractions (Fig. 5C). In contrast, downstream-migrating NT cells and KOs tended to move in a concerted fashion with the front of the cell maintaining a continuous shape over the length of the video (Fig. 5D). These cells also appeared to attach to the coverslip primarily at the uropod and display a loosely attached cell front (Movie 3).

**Fig. 5.**
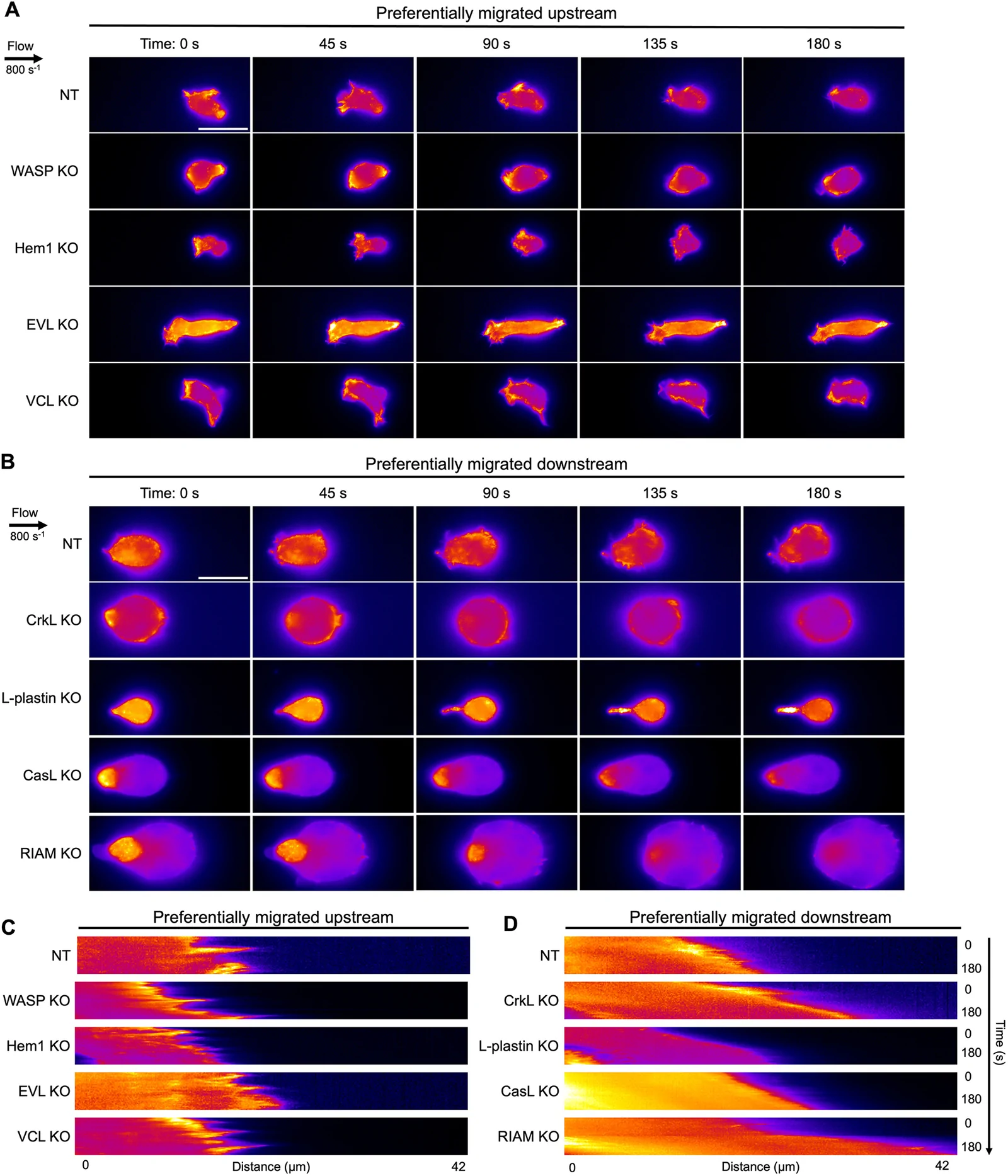
**Deleting CrkL, L-plastin, CasL and RIAM impairs F-actin localization at the leading edge.** KO cell lines expressing Lifeact–GFP were imaged by spinning-disk confocal microscopy under shear flow. Images shown are maximum intensity projections from rendered *Z*-stacks. (A) Lifeact–GFP snapshots and (C) representative kymographs of leading edges for KO cells migrating preferentially upstream, including WASP KO, Hem1 KO, EVL KO, vinculin (VCL) KO, and the subset of control NT cells migrating upstream. (B) Lifeact–GFP snapshots and (D) representative kymographs for the leading edges for KO cells migrating preferentially downstream, including CrkL KO, L-plastin KO, CasL KO and RIAM KO, and the subset of control NT cells migrating downstream. Scale bars: 25 μm (A,B). Time scale in D also applies in C. Data shown are representative of at least five cells from at least three independent experiments.

Fig. S7 shows overlays of the kymograph leading edge traces for each cell, which were analyzed to compute (1) the fraction of frames in which the movement of the leading edge was consistent with the forward direction and (2) the average velocity of the leading edge. Downstream-migrating NT cells (CrkL KO, CasL KO and RIAM KO cells) had protruding fractions that were significantly higher compared to upstream-migrating NT cells (Fig. 6A). Moreover, the leading edges of downstream-migrating NT cells and most KOs advanced with a higher velocity compared to those of upstream-migrating NT cells, though this effect only reached statistical significance for RIAM KO cells (Fig. 6B). In all, the data show that deletions in a subset of molecules that participate in LFA-1 mechanotransduction result in impaired actin localization and cytoskeletal dynamics at the leading edge.

**Fig. 6.**
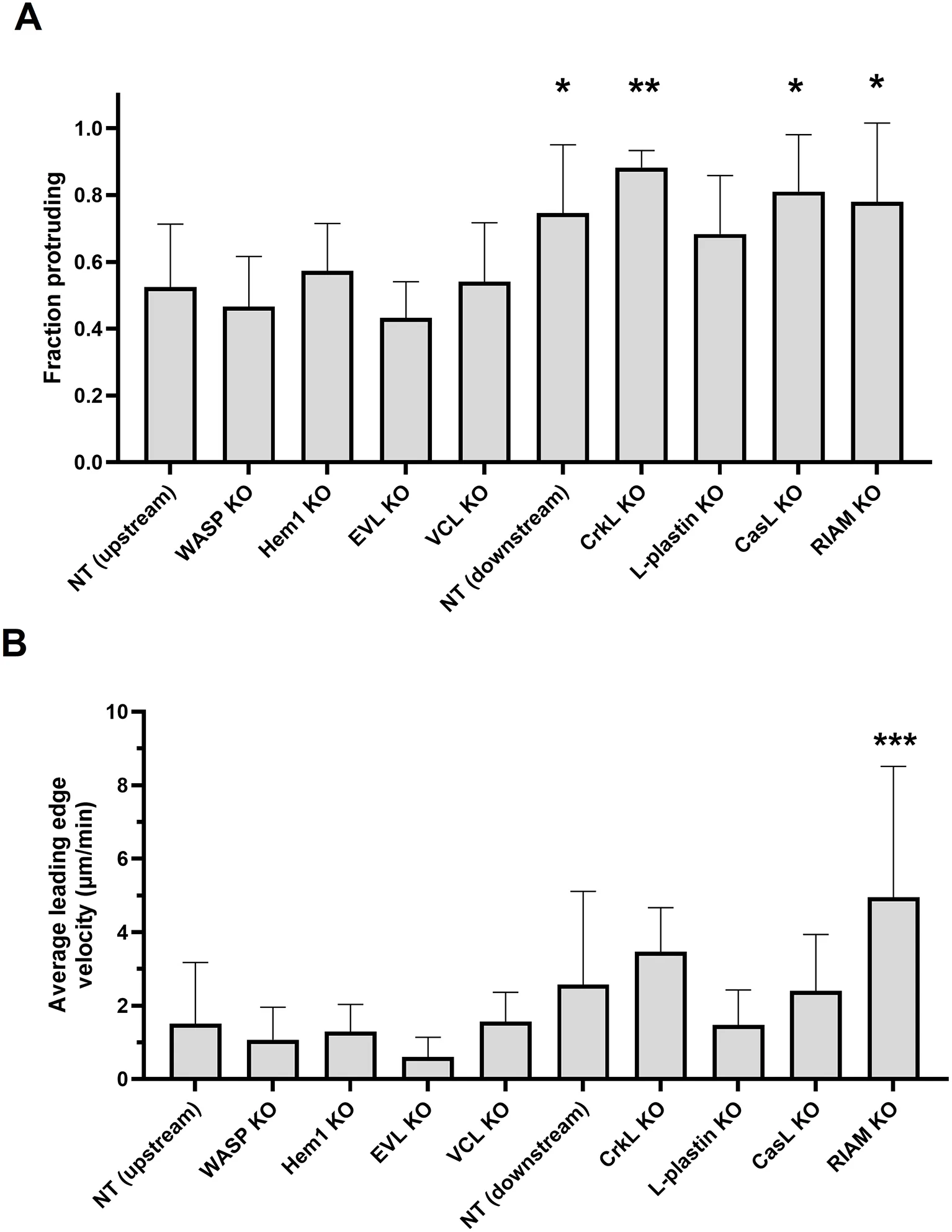
**Deletions that reverse upstream migration cause the leading edge to protrude processively.** KO cell lines expressing Lifeact–GFP were imaged under shear flow as described for Fig. 5. Kymographs of the leading edge were generated and analyzed. (A) Quantification of the fraction of time that each cell protruded over 3 min. (B) Average velocity of the leading edge. Bars represent mean±s.d., with at least five cells analyzed for each KO line. Statistics relative to NT control migrating upstream were calculated using a one-way ANOVA with Dunnett's multiple comparisons (*P<0.05; **P<0.01; ***P<0.001).

## DISCUSSION

It had previously been shown that the LFA-1 effectors, including the Crk adaptor proteins, and cCbl mediate leukocyte upstream migration on ICAM-1 under shear flow and/or F-actin localization at the leading edge (Roy et al., 2020, 2018). In this study, we investigated the role of additional LFA-1 signaling intermediates in KG-1a upstream migration, including molecules that regulate actin polymerization, link integrins to the actin cytoskeleton or participate in Crk family protein signaling. We found that CrkL, CasL, L-plastin and RIAM – all molecules that physically or biochemically link LFA-1 signaling to actin cytoskeleton responses – were required for KG-1a upstream migration. Our data support the role of shear flow-induced mechanotransduction through the integrin LFA-1 to drive the direction of immune cell migration under shear flow.

It was striking that some deletions in the CRISPR panel had no effects on random migration but had profound effects on upstream migration, whereas some deletions had profound effects on random migration but had no effects on upstream migration. This disconnect can be attributed to shear-flow induced mechanotransduction through integrins. LFA-1–ICAM-1 bonds have been shown to be catch bonds (Dembo et al., 1988; Kong et al., 2009). The applied shear force likely strengthens integrin–ligand bonds at the leading edge, leading to outside-in integrin activation and changes in integrin affinity, clustering and localization (Hogg et al., 2011). Cell signaling cascades combined with the physical linkage that integrins mediate between the substrate and cell cytoskeleton lead to changes in internal cell machinery that drive upstream migration. Our data extend the model of upstream migration proposed by Roy et al. (2020, 2018), by showing that additional LFA-1 signaling events through CasL, L-plastin and RIAM, as well as cytoskeletal responses at the leading edge are required for upstream migration. The participation of these molecules further emphasizes the importance of mechanotransduction in upstream migration. CasL is a force-sensitive scaffolding molecule involved in signaling cascades (Sawada et al., 2006), and RIAM is an adaptor protein that is key to recruiting talin and activating integrins (Chang et al., 2014). Beyond its role in bundling actin filaments, L-plastin binds to the integrin β_2_ subunit of LFA-1 and has been suggested to play a role in integrin activation as an adaptor or scaffolding protein (Jones et al., 1998; Joshi and Morley, 2022; Morley, 2012). Importantly, our results indicate that not all integrin-binding proteins play a role in shear flow-induced mechanotransduction, as vinculin expression was not required for KG-1a upstream migration. The role of vinculin in mesenchymal cell adhesion and migration is well established, where it functions to form mature focal adhesions (Carisey et al., 2013; Chorev et al., 2014; Humphries et al., 2007). Although this result was unexpected, it agrees with previous work showing that deleting vinculin in murine neutrophils does not alter cell migration under shear flow (Wilson et al., 2020). As proposed by Wilson et al., highly motile leukocytes using an amoeboid mode of motility do not generate mature focal adhesions. Although vinculin can link integrins or integrin binding partners to actin, this might play a minor role in amoeboid cell migration and hence in upstream migration.

Deleting upstream migration effectors impaired F-actin localization and leading edge dynamics – effects that were not observed in KO cells that retained the ability to migrate upstream. Our data demonstrate that mechanotransduction in response to shear flow is linked to the internal actin polymerization and migration machinery of the cell. Without such biochemical signaling coupled to sufficient force generation to overcome the strong shear flow, upstream migration is not possible. Although KG-1a CrkL KO cells migrated downstream, consistent with previous findings in Crk and CrkL double KO murine CD4^+^ T lymphocytes (Roy et al., 2020), a limitation of this study is that the experiments were conducted in a single cell line. Future KO migration studies under shear flow using primary immune cells or other immune cell lines would enable more broad generalization of our findings. In addition, it will be informative to study the role of other molecules known to be involved in mechanotransduction such as the Piezo1 ion channel (Ni et al., 2024) or the adaptor protein lamellipodin (RAPH1) (Dimchev et al., 2020), and ultimately measure spatiotemporal contractile forces generated by KO cells through traction force microscopy (Dembo and Wang, 1999; Lee and Hammer, 2026). It will also be instructive to determine how shear flow affects downstream signaling and phosphorylation through CasL, L-plastin and RIAM. Taken together, our study supports a model of upstream migration involving shear flow-induced integrin mechanotransduction, and involving signaling through LFA-1, CrkL, CasL, L-plastin and RIAM, to modulate F-actin dynamics at the leading edge.

We found that KG-1a cells lacking upstream migration effectors CrkL, CasL, L-plastin and RIAM phenotypically resembled KG-1a cells migrating downstream on VCAM-1 surfaces. These KO cells displayed strong adhesion at the uropod while the cell front appeared to be loosely attached. Previous work by Hornung et al. has presented a bistable system that determines the direction of T lymphocyte migration under shear flow (Hornung et al., 2020). In the first state, a raised, detached uropod and a firmly attached lamellipod leads to upstream migration through a ‘wind vane mechanism’, in which directional motion is guided by the hydrodynamic properties of the T lymphocyte; in the second state, a firmly attached uropod and a loosely attached lamellipod leads to downstream migration through a ‘lamellipod-focusing’ mechanism. Our Lifeact–GFP videos showing significant actin activity in the lamellipod during upstream migration are consistent with these results. Studying the localization and affinity states of LFA-1 and VLA-4 in KO cells will be helpful to reconcile the role of integrin mechanotransduction, adhesion and passive steering during upstream migration.

We found that the presence of dynamic F-actin rich lamellipodia was a hallmark of cells migrating upstream under shear flow. Our data show that the actin regulatory proteins Hem1, WASP and EVL are dispensable for formation of F-actin-rich lamellipodia and upstream migration. Although surprising, this finding builds on earlier work from our lab showing that upstream migration of other leukocytes does not require the Arp2/3 complex, PI3K or Cdc42 (Kim and Hammer, 2019; Roy et al., 2020). This counterintuitive finding may be explained by the inherent redundancy built into actin assembly mechanisms. There are three main families of actin polymerization machinery – the Arp2/3 complex, Ena/VASP and formins – which compete for the same pool of actin monomers to form branched or linear actin filaments (Thompson et al., 2022). Disrupting molecules involved in branched actin polymerization induces cells to rely more heavily on Ena/VASP or formin-mediated linear actin polymerization (Davidson et al., 2018; Rotty and Bear, 2014). We used our KG-1a KO cells and the pan-formin inhibitor SMIFH2 to explore migration under shear flow when the Arp2/3 complex and formins were perturbed. We found that upstream migration was inhibited following SMIFH2 treatment, but there were no additional effects of deleting Hem1 or WASP in addition to SMIFH2 treatment on downstream migration. These results, combined with the Lifeact–GFP videos, indicate that formins, but not the Arp2/3 complex, are key components of the upstream migration signaling machinery. Because only EVL within the Ena/VASP family proteins (Mena, EVL, VASP) was deleted and these proteins function redundantly, we cannot make conclusive statements regarding the role of Ena/VASP proteins in upstream migration. Since recent studies indicate that SMIFH2 treatment can be associated with off-target effects (Capella et al., 2025; Innocenti, 2023; Nishimura et al., 2021), future work using CRISPR/Cas9 to determine the role of individual formins, such as those in the mDia family, will provide important insights into the actin polymerization machinery that leads to KG-1a upstream migration.

Although there are reports that upstream migration is coupled to efficient transendothelial migration (Anderson et al., 2019), the subsequent step of the multi-step leukocyte adhesion cascade (Ley et al., 2007), the mechanisms through which these processes are linked is not clear. Previous *in vivo* studies have demonstrated a correlation between upstream migration effectors (Crk adaptor proteins, CasL, L-plastin and RIAM) and the ability of murine lymphocytes to cross blood endothelial barriers. Crk and CrkL-deficient T lymphocytes, CasL-deficient T lymphocytes and RIAM-deficient PMNs show impaired extravasation to reach graft-versus-host disease (GVHD) target organs (Huang et al., 2015; Klapproth et al., 2015; Kurtz et al., 2025). In addition, CasL-deficient T lymphocytes, RIAM-deficient T and B lymphocytes, and L-plastin-deficient B lymphocytes show impaired trafficking to secondary lymphoid organs such as the lymph node and spleen (Seo et al., 2005; Su et al., 2015; Todd et al., 2011). Our data extend this correlation between immune cell upstream migration and extravasation, which may both require the presence of dynamic F-actin rich lamellipodia. Further investigation of the molecules required for upstream migration using *in vitro* transendothelial migration assays will provide valuable insights into mechanisms that link upstream migration and extravasation as well as the physiological role of upstream migration.

Beyond its physiological relevance for efficient tissue patrolling and extravasation, upstream migration also provides a fertile framework to further study immune cell mechanotransduction, in which forces applied to cells lead to biochemical signals that in turn modulate cell behavior. Our findings demonstrate that signaling through LFA-1 allows leukocytes to sense shear flow and guides two-dimensional migratory responses on the apical surface of the endothelium under shear flow. By performing traction force microscopy, we have recently shown that under flow conditions on ICAM-1 surfaces, KG-1a WT cells respond to the applied force by exerting even larger traction forces to migrate against the direction of flow (Lee and Hammer, 2026). In this interesting manifestation of mechanotransduction, a force leads to biochemical signaling, which in turn leads to greater cell-generated forces. In future work, we will explore how the deletions described in this paper affect the ability of cells to generate traction forces.

## MATERIALS AND METHODS

### Antibodies and reagents

Recombinant human ICAM-1 Fc chimera and VCAM-1 Fc chimera were obtained from R&D Systems (Minneapolis, MN, USA). For pharmacological inhibition, DMSO (Cell Signaling Technologies, Danvers, MA, USA) and SMIFH2 (Sigma, St. Louis, MO, USA) were used. SMIFH2 was stored frozen at stock concentrations of 25 mM in DMSO. Anti-GAPDH clone 6C5 was obtained from Millipore Sigma (St. Louis, MO, USA). Anti-CrkL (sc-319) was from Santa Cruz (Dallas, TX, USA). Anti-LCP1 (5350), anti-WASP (4271), anti-WAVE2 (3659), anti-EVL (12536), anti-CasL (4044) and anti-vinculin (4650) were obtained from Cell Signaling Technologies. Anti-NCKAP1L (Hem1; ab272631) and anti-RIAM (ab92537) were purchased from Abcam (Cambridge, UK). Antibodies for flow cytometry were obtained from BioLegend (San Diego, CA, USA) and used at 1:100 dilution for mouse anti-CD11a FITC (clone HI111), anti-CD18 PE (clone TS1/18), anti-CD49d (clone 9F10) and anti-CD29 PE/Cy7 (clone TS2/16). All antibodies were validated by the manufacturer.

### Cell culture

KG-1a cells (ATCC CCL-246.1, Manassas, VA, USA) were cultured in IMDM with Glutamax (Gibco 31980030, Grand Island, NY, USA) containing 10% fetal bovine serum (FBS; Sigma F2442), and penicillin and streptomycin (Gibco 15140122). HEK293T cells were cultured in DMEM containing high glucose (Gibco 11965092), 10% FBS, penicillin and streptomycin. All cells were maintained at 37°C and 5% CO_2_.

### Generation of knockout cell lines

CRISPR/Cas9 KO gRNA sequences were designed using CHOPCHOP and UCSC Genome Browser CRISPR tool (Table S1) (Labun et al., 2019; Perez et al., 2025). Oligonucleotides and primers were ordered from IDT (Newark, NJ, USA). Guide sequences were cloned into the pSpCas9(BB)-2A-GFP (px458) vector (Addgene plasmid 48138) as described previously (Ran et al., 2013). KG-1a cells were electroporated with 1 μg of plasmid using the Neon Transfection kit (Thermo Fisher) using manufacturer's settings (Gundry et al., 2016). 48–72 h following transfection, GFP^+^ cells were isolated using fluorescence-activated single cell sorting into 96-well plates containing cell culture medium using FACSJazz (BD Biosciences, San Jose, CA, USA). Clonal cell lines were validated by genomic DNA sequencing and western blotting. Statistically significant findings were confirmed with independently derived clones.

### Generation of Lifeact–GFP cells

The Lifeact–GFP lentiviral transfer plasmid pLX301-Blast-Lifeact-mEGFP with Blasticidin resistance was described previously (Comrie et al., 2015). HEK293T cells were transfected with pLX301-Blast-Lifeact-mEGFP, pMD2.G (Addgene plasmid 12259) and psPAX2 (Addgene plasmid 12260) as described previously using Lipofectamine 3000 (Life Technologies, Waltham, MA, USA) (Sanjana et al., 2014). Cell medium was replaced after 6–8 h, and lentiviral supernatant was collected at 24, 48 and 72 h post transfection. The filtered supernatant was concentrated using an Amicon Ultra 100 kDa cutoff centrifugal filter (Millipore Sigma). KG-1a cells were mixed with the concentrated lentiviral supernatant in cell culture medium containing 8 μg ml^−1^ polybrene (Millipore Sigma) then spinfected at 1000 ***g*** at 37°C for 1.5–2 h. Following spinfection, cell medium was replaced with complete medium, and transduced cells were placed into the cell culture incubator. 72 h following transduction, 5 μg ml^−1^ Blasticidin (Gibco) was added to select for transduced cells.

### Western blotting

For western blotting, 1×10^6^ cells were lysed with 1× cold cell lysis buffer (Cell Signaling Technology, 9803) supplemented with EDTA-free protease inhibitor cocktail (Roche, Indianapolis, IN, USA) as described previously (Roy et al., 2020). Lysates were incubated on ice and vortexed every 10 min. After 20 min, lysates were centrifuged for 10 min at 13,000 rpm using the FA-24x2 rotor (Eppendorf, Enfield, CT, USA) at 4°C. Lysates were mixed with NuPAGE 4× LDS sample buffer (Invitrogen) containing dithiothreitol (DTT; 50 mM final) and heated at 95°C for 5 min. Proteins were separated by SDS-PAGE using the Invitrogen Novex Mini-cell system with NuPAGE 4–12% BisTris gradient gels. Proteins were transferred to 0.2 μm nitrocellulose membranes (Invitrogen) and blocked using Blocking Buffer (LI-COR, Lincoln, NE, USA) mixed 1:1 with TBS for 1 h. Except for anti-GAPDPH (1:5000 dilution), primary antibodies were prepared at 1:1000 dilution in 1× TBS containing 0.1% Tween-20 (TBST) with 2% bovine serum albumin (BSA). Primary antibodies were incubated with membranes overnight on a shaker at 4°C. After washing membranes three times with 1× TBST, membranes were incubated with fluorophore-conjugated secondary antibodies purchased from LI-COR. After washing membranes three times with 1× TBST, membranes were imaged using a LI-COR Odyssey M imaging system. Blot transparency images are provided in Figs S8 and S9.

### Migration of KG-1a cells

Surfaces for cell migration were functionalized by coating a plastic microscope slide (1″×3″; Electron Microscopy Sciences, Hatfield, PA, USA) or the μSlide LuerI^0.2^ chamber (ibidi, Gräfelfing, Germany) with 2 μg ml^−1^ Protein A/G (Thermo Fisher Scientific, Waltham, MA, USA) overnight at 4°C as described previously (Mittal et al., 2024). After washing the surface with PBS, non-specific binding was blocked with 0.2% Pluronic F-127 (Sigma-Aldrich) for 30 min. Finally, surfaces were coated with 5 μg ml^−1^ of recombinant human ICAM-1 Fc chimera overnight at 4°C. Following assembly, the flow chamber was flushed with medium before the addition of cells to the chamber. Cells were allowed to adhere to the surfaces for 30 min, and running medium was injected into the flow chamber using a syringe pump (Harvard Apparatus, Holliston, MA, USA) to establish indicated shear rates (Dominguez et al., 2015). For pharmacological inhibition, cells were treated with 5 μM SMIFH2 or DMSO (control) in running medium for 30 min at 37°C and 5% CO_2_. Cells were then injected into a flow chamber as described above. Running medium also contained the respective concentrations of the pharmacological inhibitor.

All timelapse images were acquired in the microscopy software MetaMorph (Molecular Devices, San Jose, CA, USA). When the Nikon (Melville, NY, USA) Eclipse TE300 phase contrast microscope with a 10×/NA 0.25 objective and QImaging Retiga EXi Fast camera (Teledyne Technologies, Thousand Oaks, CA, USA) was used to characterize migration (Figs 1–4; Figs S1–S5), images were acquired every minute for 30 min. Cells were kept at 37°C and 5% CO_2_ at all times, and IMDM containing 5% FBS was used as the running medium. To assess blebbing frequency, higher magnification images were acquired every 1–2 s for 1 min with a 40×/NA 0.55 objective (Fig. S6C). To acquire DIC cell morphology images (Fig. S6A,B, Movie 1) and Lifeact videos (Fig. 5; Movies 2,3), the Leica (Wetzlar, Germany) DMi8 inverted microscope with a confocal spinning-disk unit (Spectral Applied Research, Richmond Hill, Canada) with a 63×/NA 1.0 objective and Hamamatsu ORCA-Flash 4.0 V2 digital sCMOS camera (Shizuoka, Japan) was used. For morphology videos, images of migrating cells were acquired every second for 1 min. For the Lifeact–GFP videos, nine *z*-stacks with a 0.25 µm step size were taken every 5 s for 3 min. Cells were kept at 37°C, and Leibovitz's L-15 medium (Gibco 11415064 or Gibco 21083027) was used as the running medium.

### Cell tracking and analysis

For videos of migrating cells taken at 10×, cells were segmented with a custom-trained model in Cellpose (Pachitariu and Stringer, 2022; Stringer et al., 2021), then cell tracks were analyzed in Trackmate using Fiji (Schindelin et al., 2012; Tinevez et al., 2017). Cells that remained in the field of view during the length of the experiment that moved at least two cell lengths were included in the analysis. Cell tracks were analyzed using a custom script in MATLAB (The MathWorks, Natick, MA, USA) to calculate speed, random motility coefficient (μ), and persistence time by calculating the mean squared displacement of each cell fitted to the Dunn's equation as described previously (Dunn, 1983; Hind et al., 2014). The migration index (MI) is defined as the ratio of the net *x*-displacement of the cell to the total cell displacement and ranges from a value of −1 to 1. A negative MI represents upstream migration, and positive MI represents downstream migration. Directionality is defined as net displacement divided by the total distance traveled, where values closer to 1 represent migration in a straight path.

### Analysis of migrating Lifeact–GFP cells

In ImageJ, *z*-stacks from each timepoint were turned into a timelapse series of maximum intensity projections. A kymograph was created by drawing a line starting from the centroid of the cell at the first frame towards the direction of migration. The kymograph image was filtered using a Gaussian blur (sigma=1), binarized, then the ‘Find Edges’ function was used to detect the leading edge of the cell. In MATLAB, overlays of the normalized distance the cell front traveled relative to the initial position, average line and standard deviation were plotted. In addition, the percentage of time that each cell was protruding over 3 min and the average velocity of the cell front were computed.

### Flow cytometry

Fc receptors were blocked using Human TruStain FcX (Biolegend), then cells were stained with Live/Dead Aqua (Invitrogen) in PBS following the manufacturer's protocol. Cells were stained with antibodies in 1% BSA in PBS for 30 min at room temperature. Flow cytometry was performed using an Aurora spectral analyzer (Cytek Biosciences, Fremont, CA, USA), and data were analyzed using FlowJo (BD Biosciences) using a gating strategy similar to that previously described (Roy et al., 2020), where cells were gated first by size and granularity to exclude debris, followed by singlet gating and viability gating. To quantitively compare integrin expression levels, the median fluorescence intensity was compared (*n*=3).

### Statistical analysis

All data are presented as mean±s.d. Statistical analysis was conducted in GraphPad Prism 10 (GraphPad, San Diego, CA, USA). When only two groups were compared, an unpaired, two-tailed *t*-test was used. When more than two groups were compared, a one-way ANOVA with post-hoc Dunnett's multiple comparisons was used. *P*<0.05 was considered significant. *P<0.05; **P<0.01; ***P<0.001.

## Supplementary Material

10.1242/joces.265036_sup1 Supplementary information
