# Supplementary material for: Mechanotransduction through LFA-1 controls upstream migration in the KG-1a stem cell line
Source: J Cell Sci. 2026 Oct 14;139(19):jcs265036. doi: 10.1242/jcs.265036 (PMC13741953; doi:10.1242/jcs.265036)
Supplement: Supplementary file 1 [file joces-139-265036-s1.pdf]

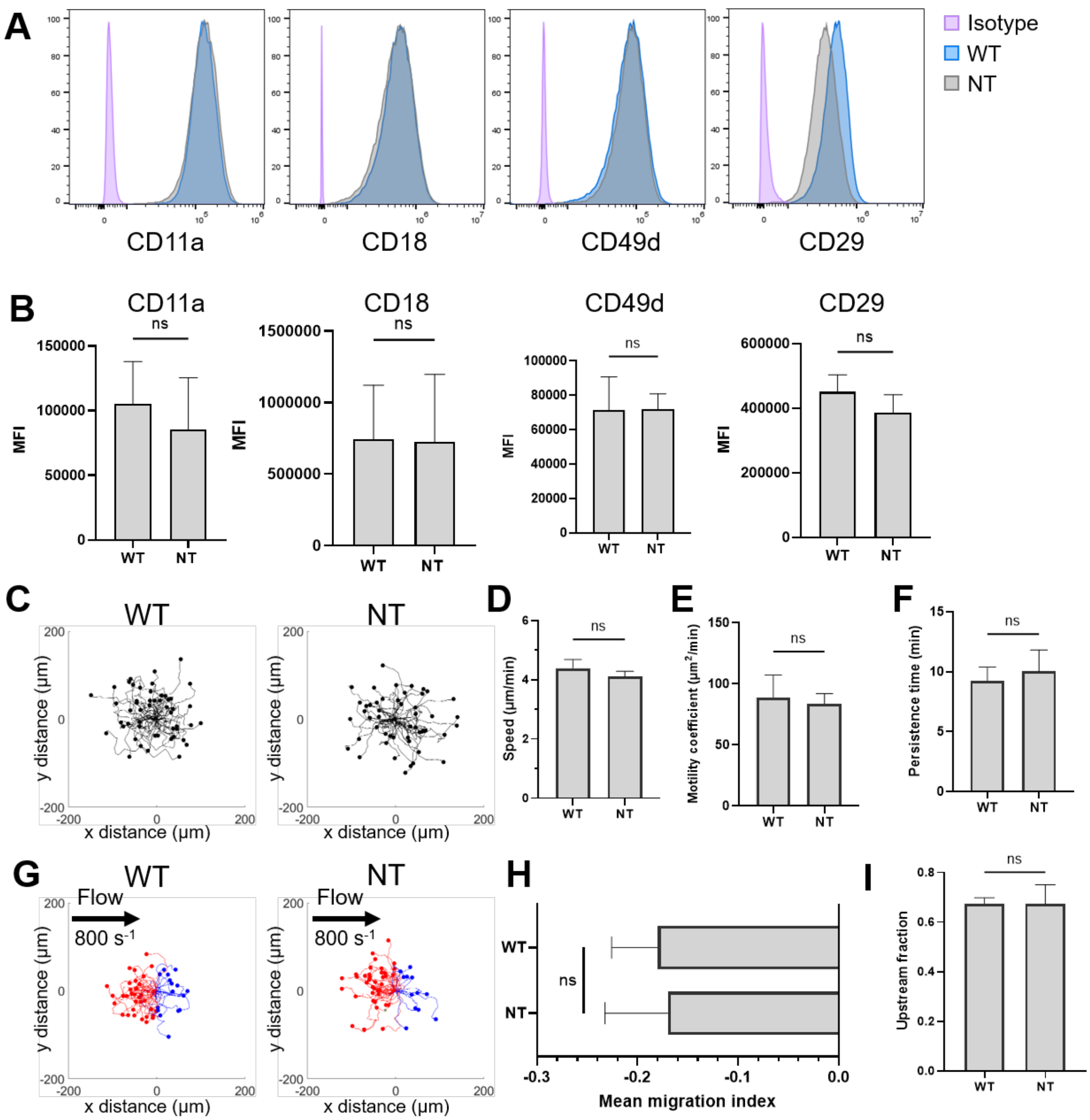

Fig. S1. Negative control KG-1a cells transfected with CRISPR/Cas9 non-targeting (NT) guide RNA migrate similar to WT

A) Cell surface integrin expression levels for CD11a, CD18, CD49d, and CD29 for KG-1a WT and KG-1a NT cells were assessed by flow cytometry. B) Median fluorescence intensity levels of respective integrin subunits were compared for WT and NT cells with 3 independent samples. Bars represent mean ± s.d. C) Representative plots of KG-1a WT and NT cells migrating on ICAM-1 in the absence of shear flow. D) Speed, E) Random motility coefficient, and F) Persistence time were calculated. G) Representative plots of KG-1a WT and NT cells

Journal of Cell Science • Supplementary information

migrating on ICAM-1 surfaces under 800 s^{-1} shear flow (left to right). Red tracks indicate cells migrating upstream against the direction of flow, while blue tracks indicate cells migrating downstream with the direction of flow. H) Mean migration index and I) Fraction of cells migrating upstream were quantified. D-F and H-I: Bars represent mean ± s.d. using 3 independent experiments with at least 30 cells analyzed per experiment. Statistics were calculated using a t-test.

Journal of Cell Science • Supplementary information

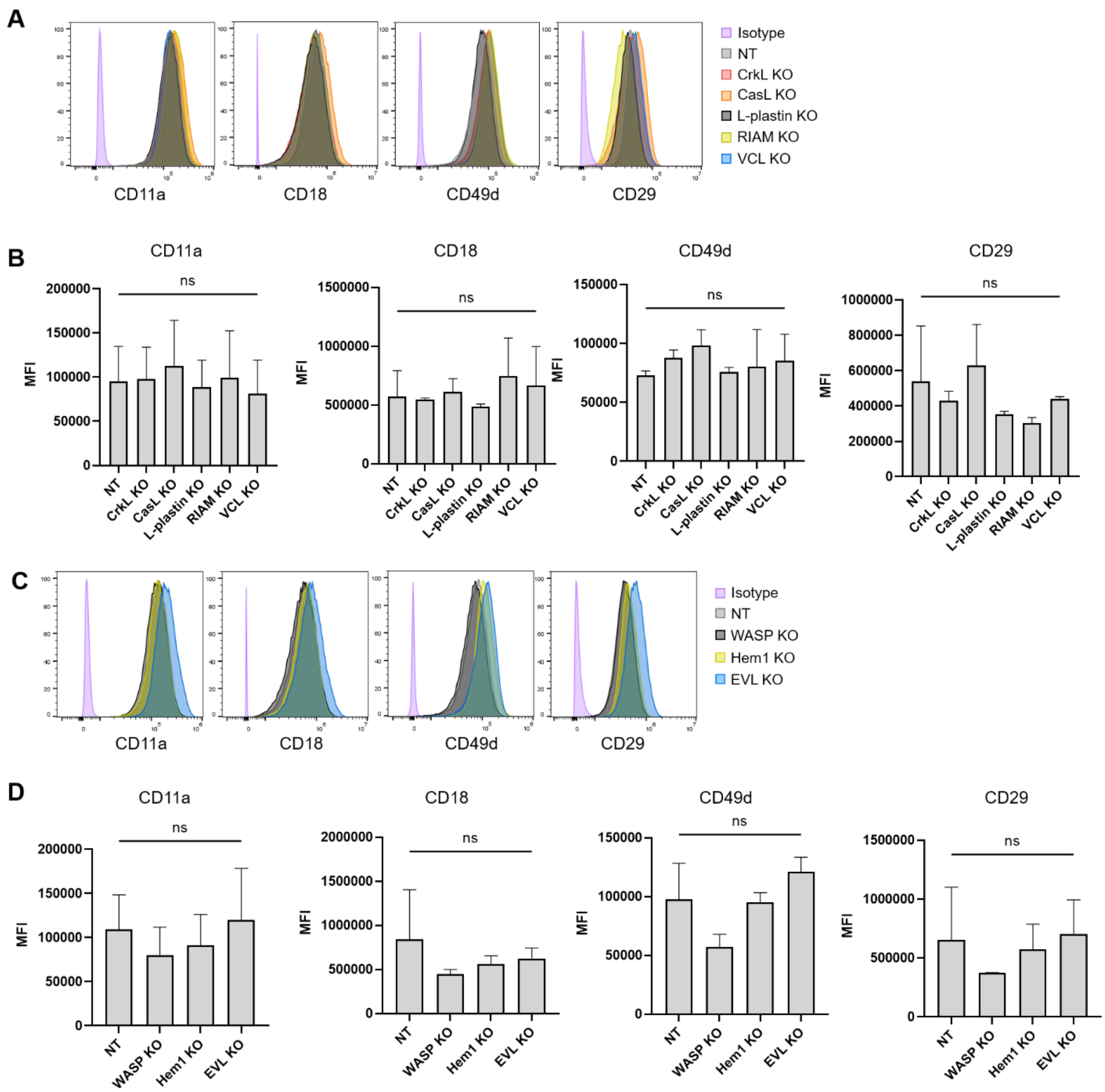

Fig. S2. CRISPR/Cas9 deletions did not alter integrin cell surface expression levels. Cell surface integrin expression levels for CD11a, CD18, CD49d, and CD29 were assessed by flow cytometry for A) NT, CrkL KO, CasL KO, L-plastin KO, RIAM KO, and VCL KO cells and C) NT, WASP KO, Hem1 KO, and EVL KO cells. B,D) Median fluorescence intensity levels of respective integrin subunits were compared with 3 independent samples. Bars represent mean ± s.d. using 3 independent samples. Statistics relative to NT control were calculated using a one-way ANOVA with Dunnett's multiple comparisons.

Journal of Cell Science • Supplementary information

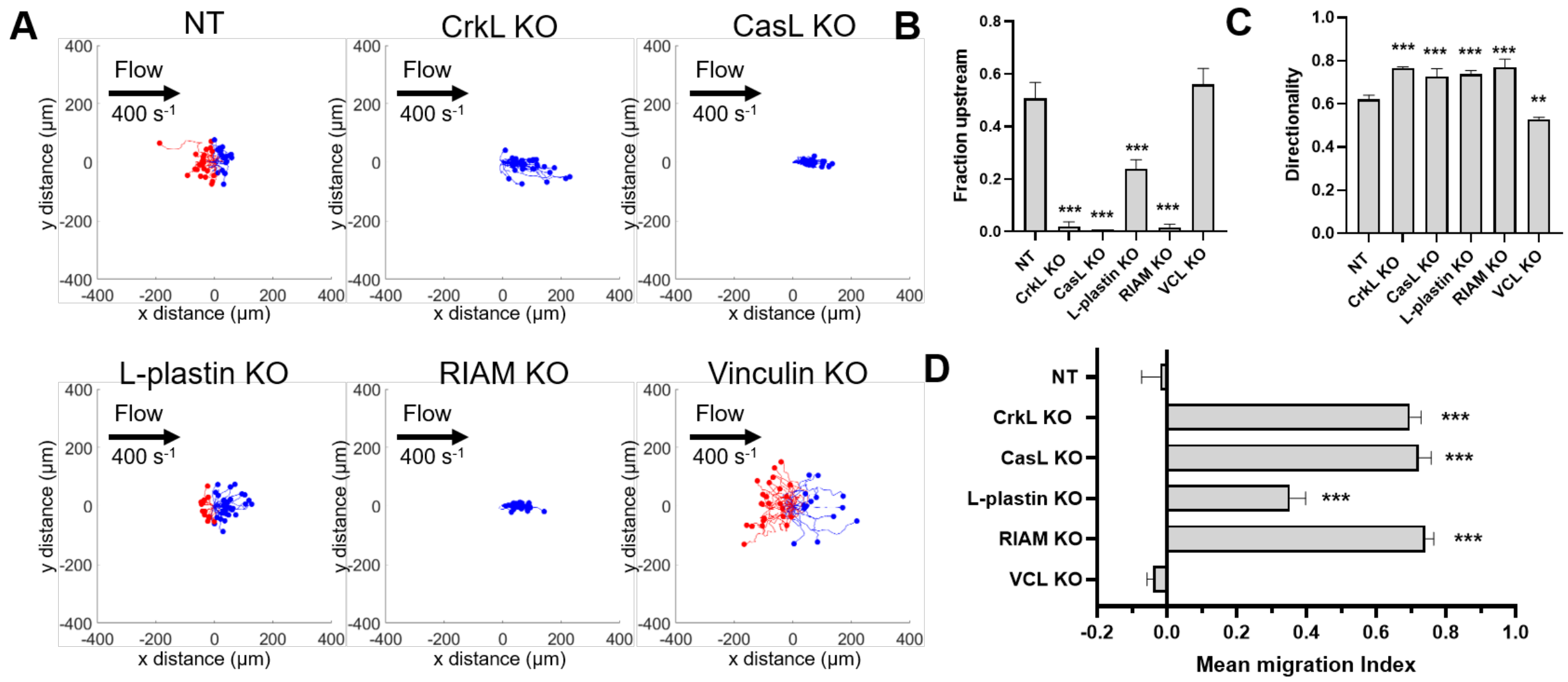

Fig. S3. KG-1a CrkL, CasL, L-plastin, and RIAM knockout cells migrate downstream on ICAM-1 at 400 s^{-1}

A) Representative plots of the KG-1a NT, CrkL KO, CasL KO, LCP1 KO, RIAM KO, and VCL KO cells migrating under 400 s^{-1} shear flow (left to right). Red tracks indicate cells migrating upstream against the direction of flow, while blue tracks indicate cells migrating downstream with the direction of flow. Migration characteristics were quantified, including the B) Fraction of cells migrating upstream, C) Mean directionality, and D) Mean migration index. B-D: Bars represent mean ± s.d. using 3 independent experiments with at least 30 cells analyzed per experiment. Statistics relative to NT control were calculated using a one-way ANOVA with Dunnett's multiple comparisons (**P<0.01; ***P<0.001).

Journal of Cell Science • Supplementary information

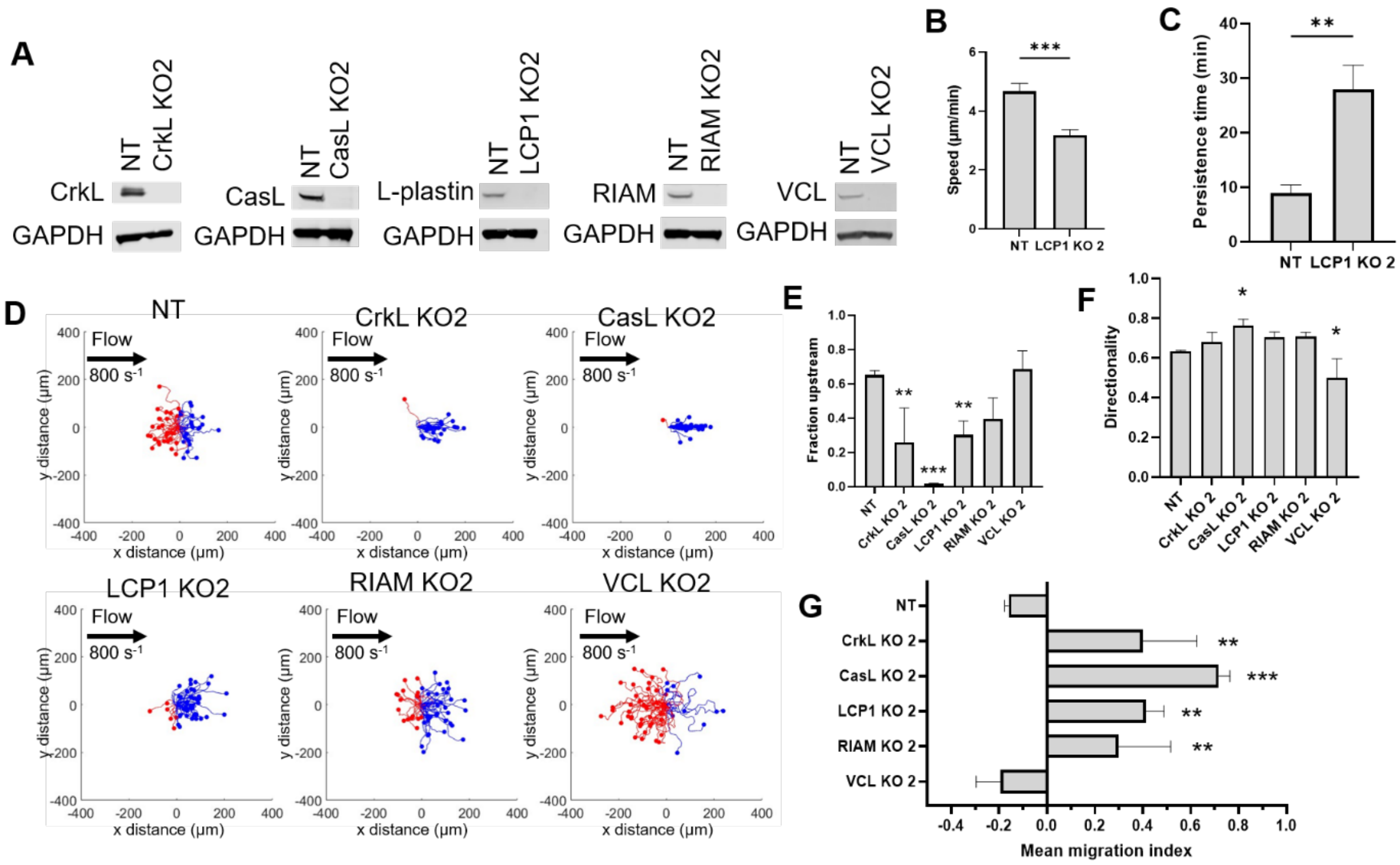

Fig. S4. Deleting CrkL, L-plastin, CasL, and RIAM consistently reverse the direction of migration in KG-1a cells.

A) Independently derived clones of CrkL KO, L-plastin (LCP1) KO, CasL KO, VCL KO, and RIAM KO were validated by Western blotting. NT, non-targeting gRNA control. GAPDH was used as a loading control. Statistically significant trends in random migration from Fig. 1 were confirmed by measuring B) random speed and C) persistence time. D) Representative plots of the independently derived clones migrating on ICAM-1 surfaces under 800 s-1 shear flow (left to right). Red tracks indicate cells migrating upstream against the direction of flow, while blue tracks indicate cells migrating downstream with the direction of flow. Migration characteristics were quantified, including the E) Fraction of cells migrating upstream, F) Mean directionality, and G) Mean migration index. B-C, E-G: Bars represent mean ± s.d. using 3 independent experiments with at least 30 cells analyzed per experiment. Statistics relative to NT control were calculated using a one-way ANOVA with Dunnett's multiple comparisons (*P<0.05; **P<0.01; ***P<0.001).

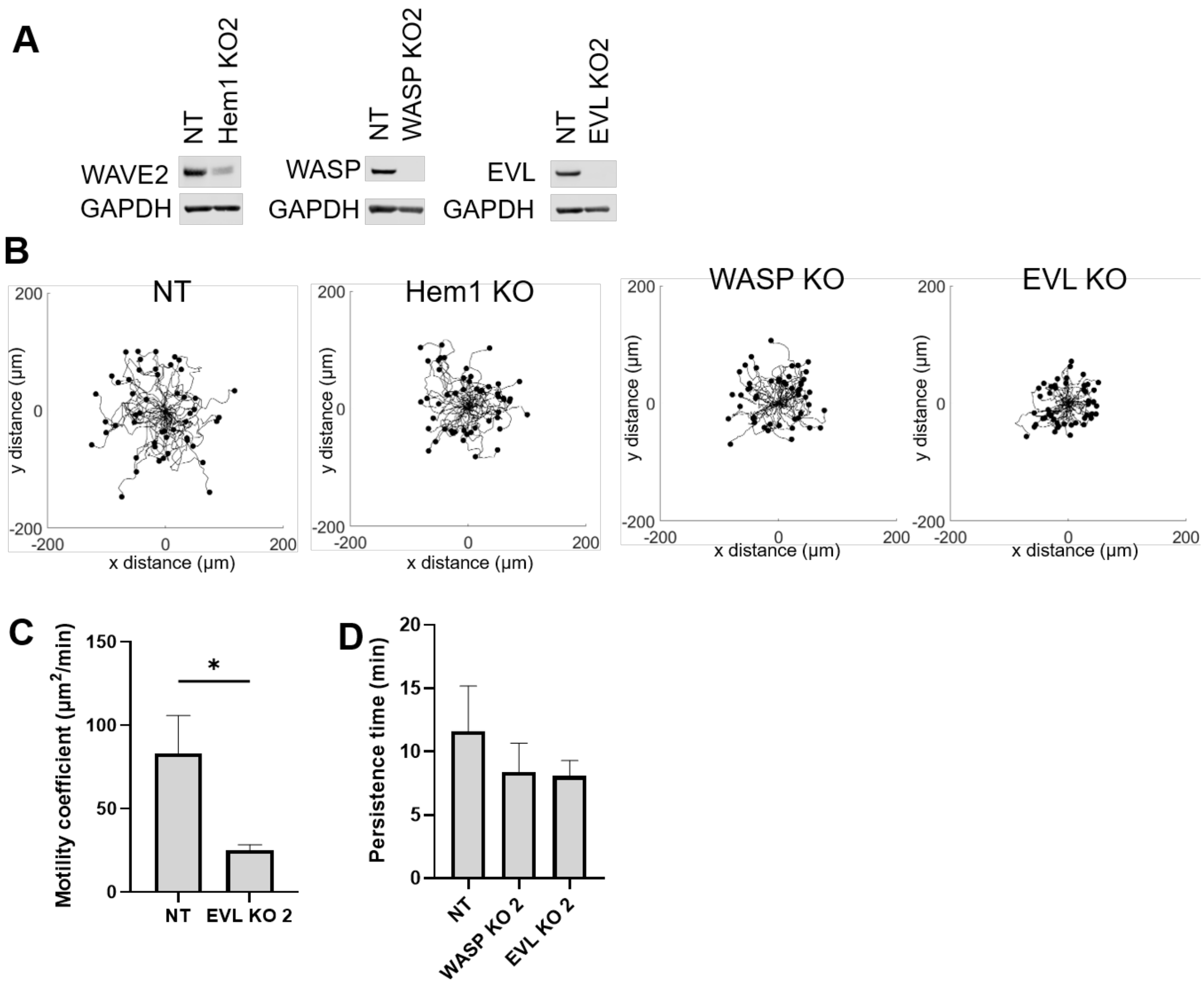

Fig. S5. Deleting EVL and WASP KO consistently affect random motility.

A) Independently derived clones of Hem1 KO, WASP KO, and EVL KO were validated by Western blotting. NT, non-targeting gRNA control. GAPDH was used as a loading control. B) Random migration on ICAM-1 surfaces was assessed, and representative cell tracks are shown. Statistically significant trends in random migration from Fig. 3 were confirmed by measuring C) random motility coefficient and D) persistence time. C,D: Bars represent mean ± s.d. using 3 independent experiments with at least 30 cells analyzed per experiment. Statistics relative to NT control were calculated using a t-test or one-way ANOVA with Dunnett's multiple comparisons (*$P<0.05$).

Journal of Cell Science • Supplementary information

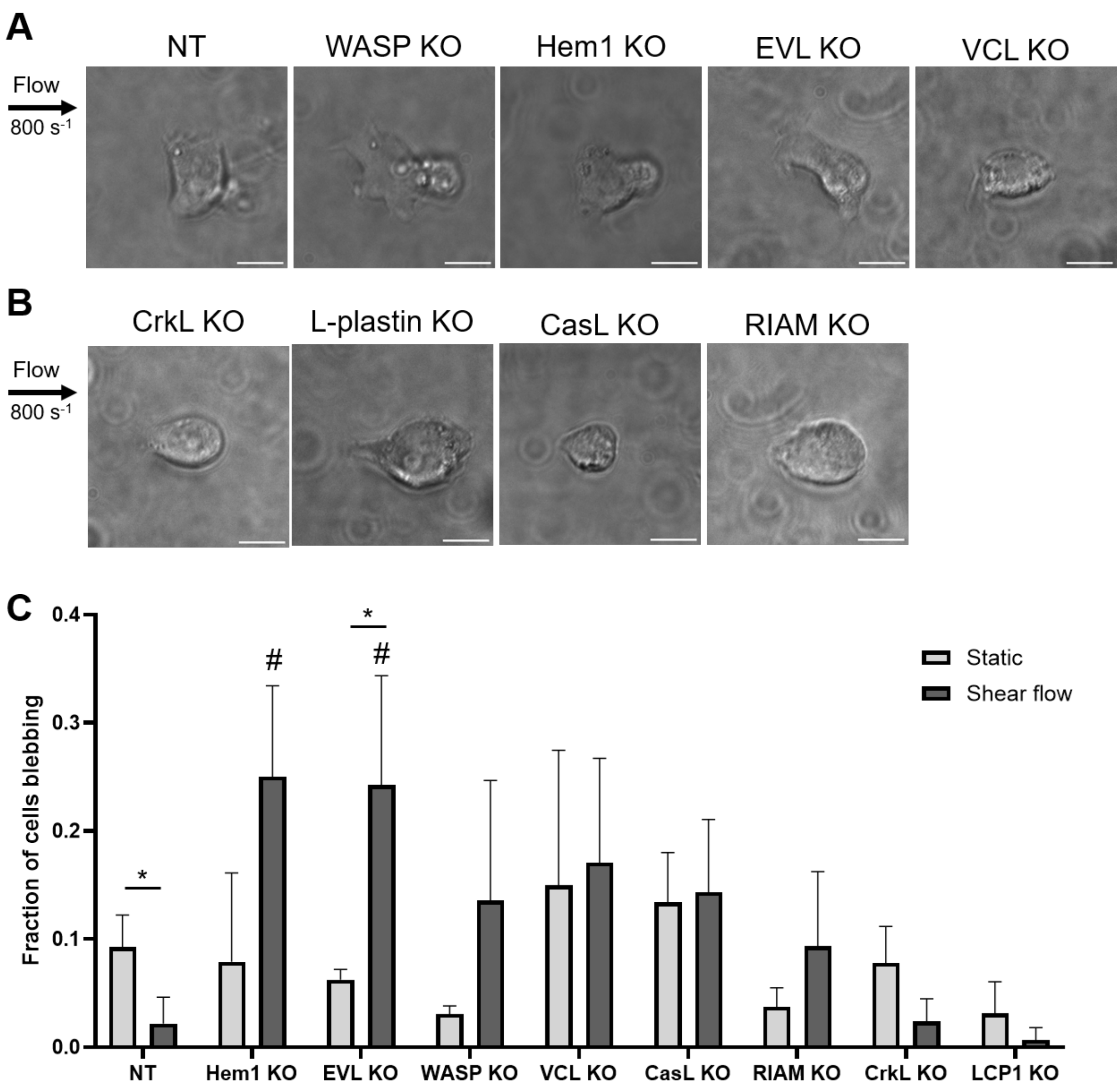

Fig. S6. Knockouts that migrate upstream display distinct cell morphologies compared to knockouts that migrate downstream.

A,B) Representative DIC images of migrating cells on ICAM-1 under 800 s^{-1} shear flow (left to right) for the subset of knockout cells migrating preferentially A) upstream and B) downstream. Scale bars are 10 µm. C) The percentage of cells that displayed a blebbing morphology was quantified. The percentage was assessed on ICAM-1 surfaces both under random migration (no shear flow) and under the influence of 800 s^{-1} shear flow. Bars represent mean ± s.d. using 3 independent experiments with at least 25 cells analyzed per experiment. C: Statistics between static and shear flow groups were calculated using a t-test (*P<0.05). Statistics within static or shear flow groups relative to NT control were calculated using a one-way ANOVA with Dunnett's multiple comparisons (#P<0.05).

Journal of Cell Science • Supplementary information

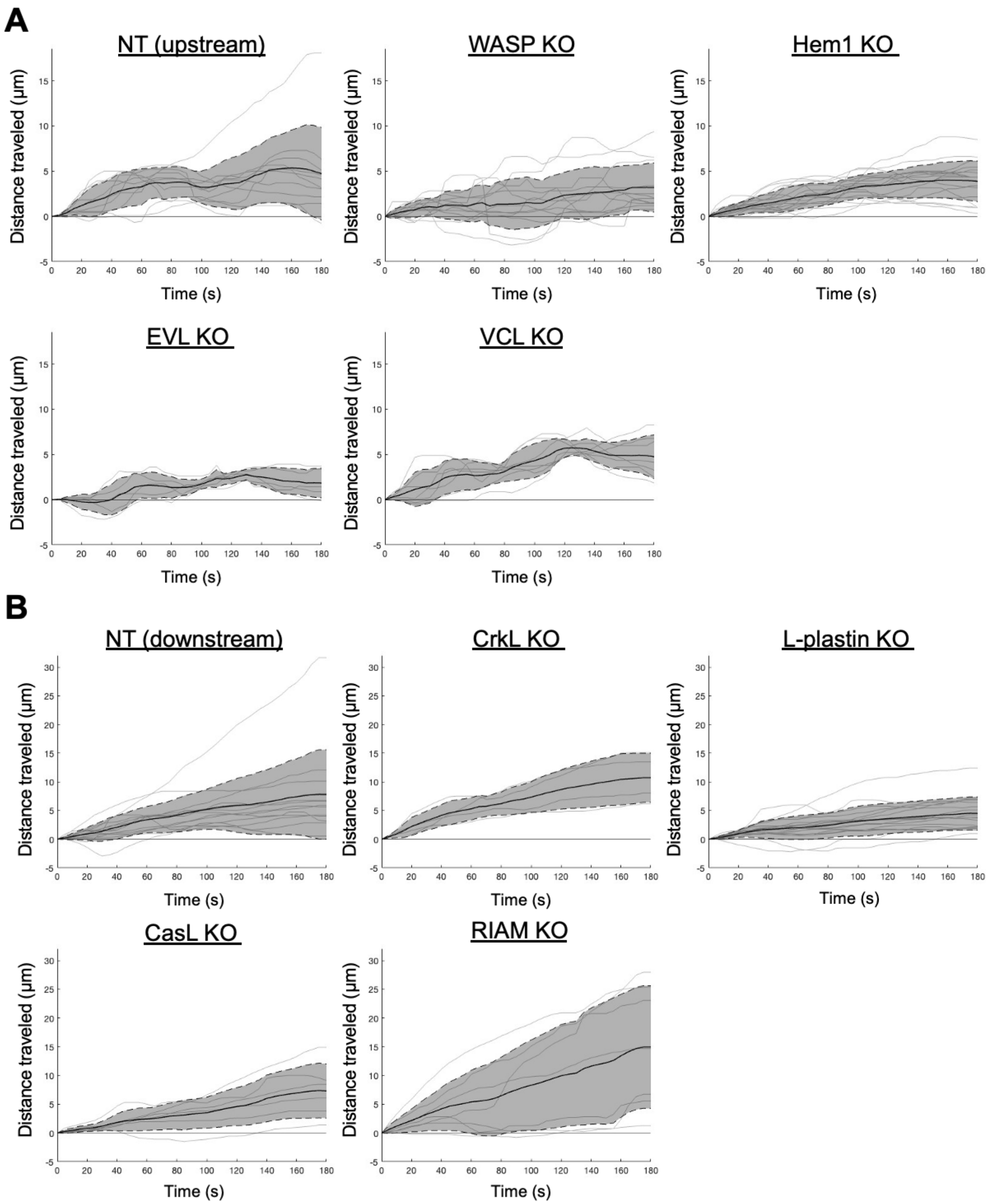

Fig. S7. Overlays of leading edge kymograph traces

Overlay of the leading edges kymograph traces from Lifeact-GFP videos for the A) subset of KG-1a mutants migrating preferentially upstream and B) subset of KG-1a mutants migrating preferentially downstream over 3 min. Each grey line represents one cell, the average line is represented by the solid black line, and the s.d. at each timepoint is represented by the shaded area. At least 5 cells were analyzed for each KO.

Journal of Cell Science • Supplementary information

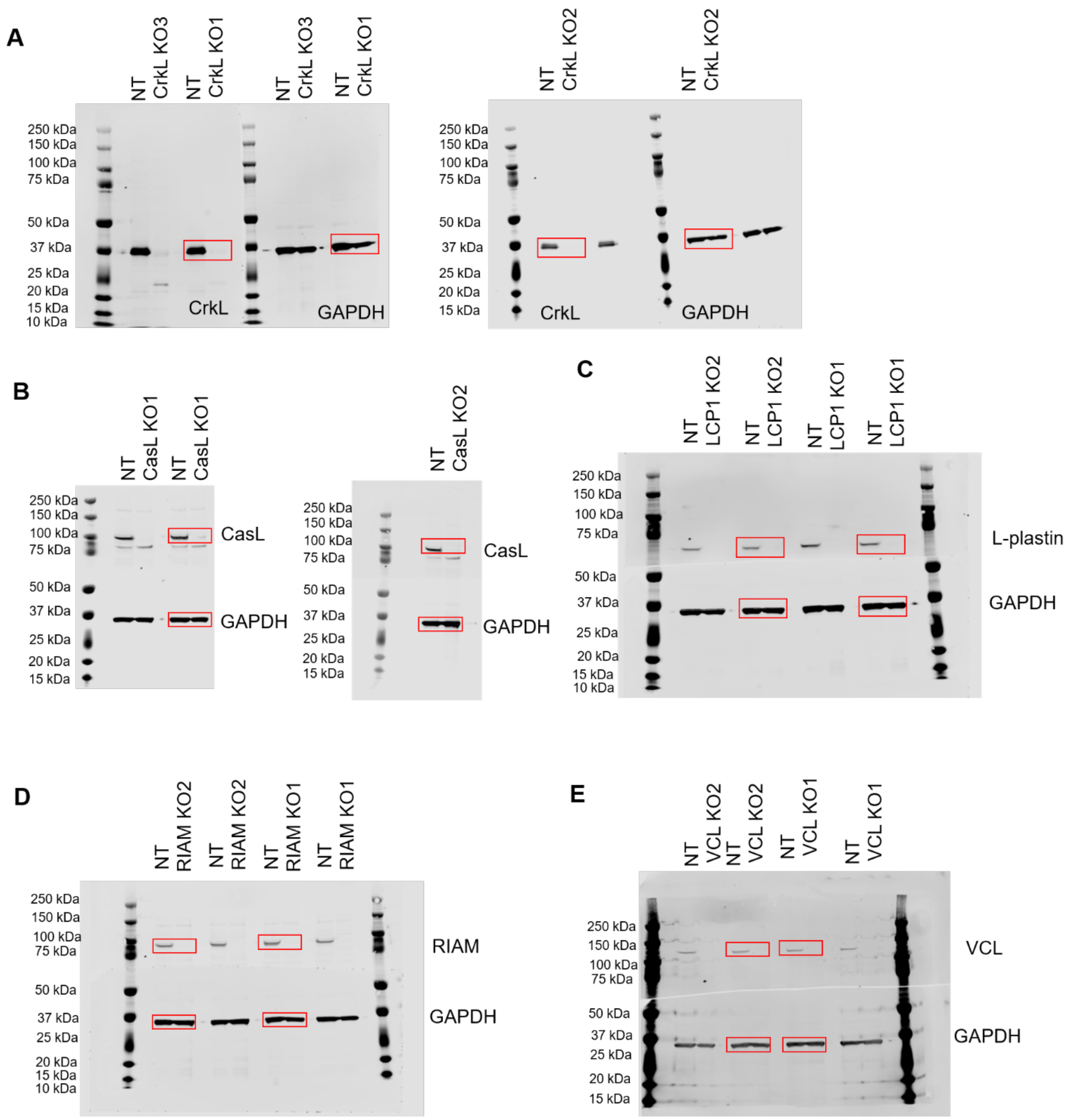

Fig. S8. Blot transparency 1

A) CrkL KO blot images for Fig. 1A and Fig. S4A. B) CasL KO blot images for Fig. 1A and Fig. S4A. C) L-plastin (LCP1) KO blot images for Fig. 1A and Fig. S4A. D) RIAM KO blot images for Fig. 1A and Fig. S4A. E) VCL KO blot images for Fig. 1A and Fig. S4A.

Journal of Cell Science • Supplementary information

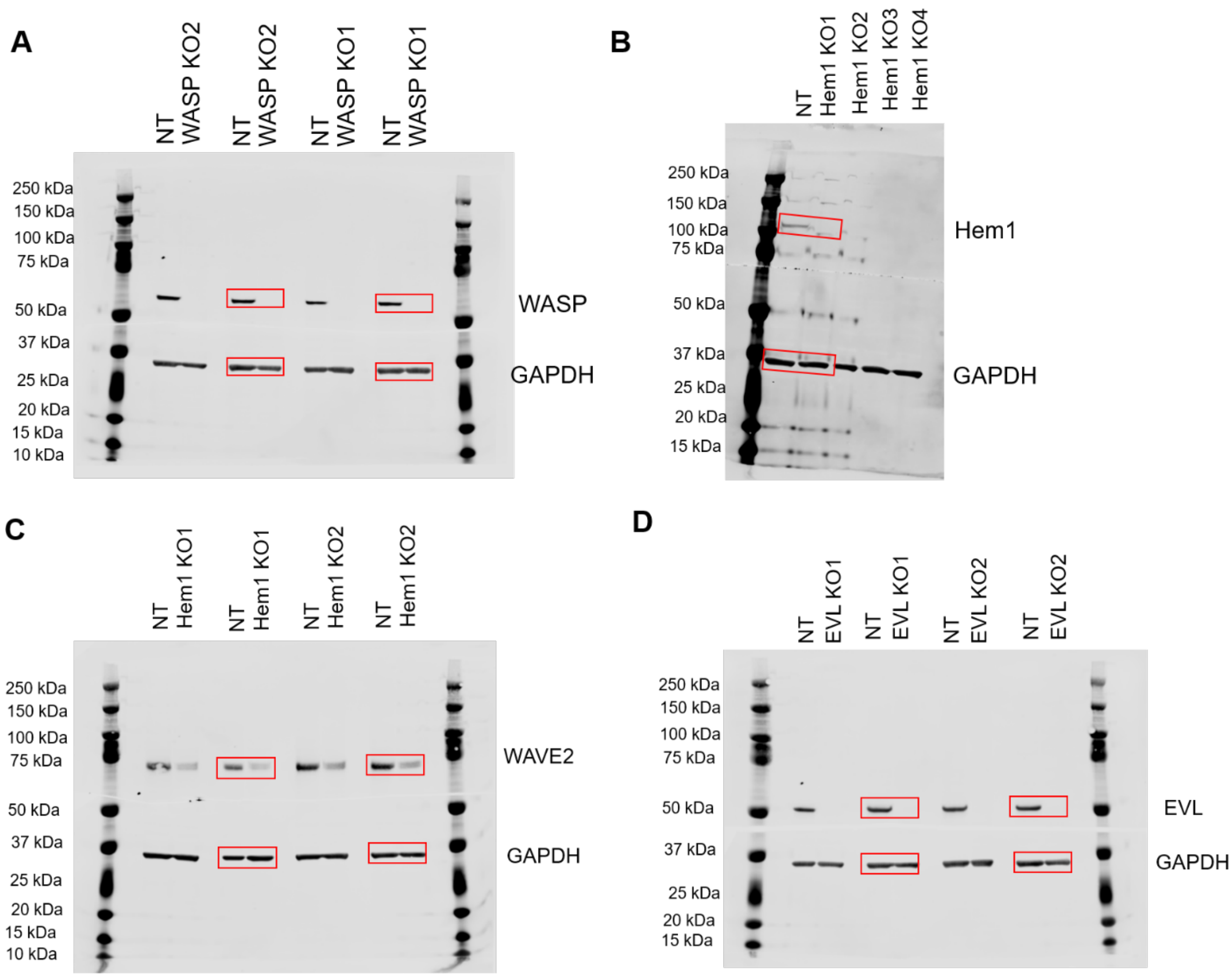

Fig. S9. Blot transparency 2

A) WASP KO blot images for Fig. 3A and Fig. S5A. B) Hem1 KO blot images for Fig. 3A. C) WAVE2 KO blot images for Fig. 3B and Fig. S5A. D) EVL KO blot images for Fig. 3A and Fig. S5A.

Journal of Cell Science • Supplementary information

Table S1. List of guide RNA sequences used for CRISPR/Cas9 gene knockout.

**Gene**	**guide RNA sequence (5’ -> 3’)**
Non-targeting (NT)	GTATTACTGATATTGGTGGG
Hem1/Nckap1l	GTCAACGGCGGCAATTTCTG
WASP	GAACTTTGCAGACGAGGACG
EVL	GGTGTCATCGTAGACCATCA
CrkL	CAACCGCCGTTTTAAGATCG
L-plastin (LCP1)	GAAGGGATTTGTGCAATCGG
CasL/Nedd9	ATATCCGTAACACCGCCAGT
Vinculin (VCL)	ATCGTGCGCGTATGAAACAC
RIAM	AAGGTTGTCCAGAACATCTC

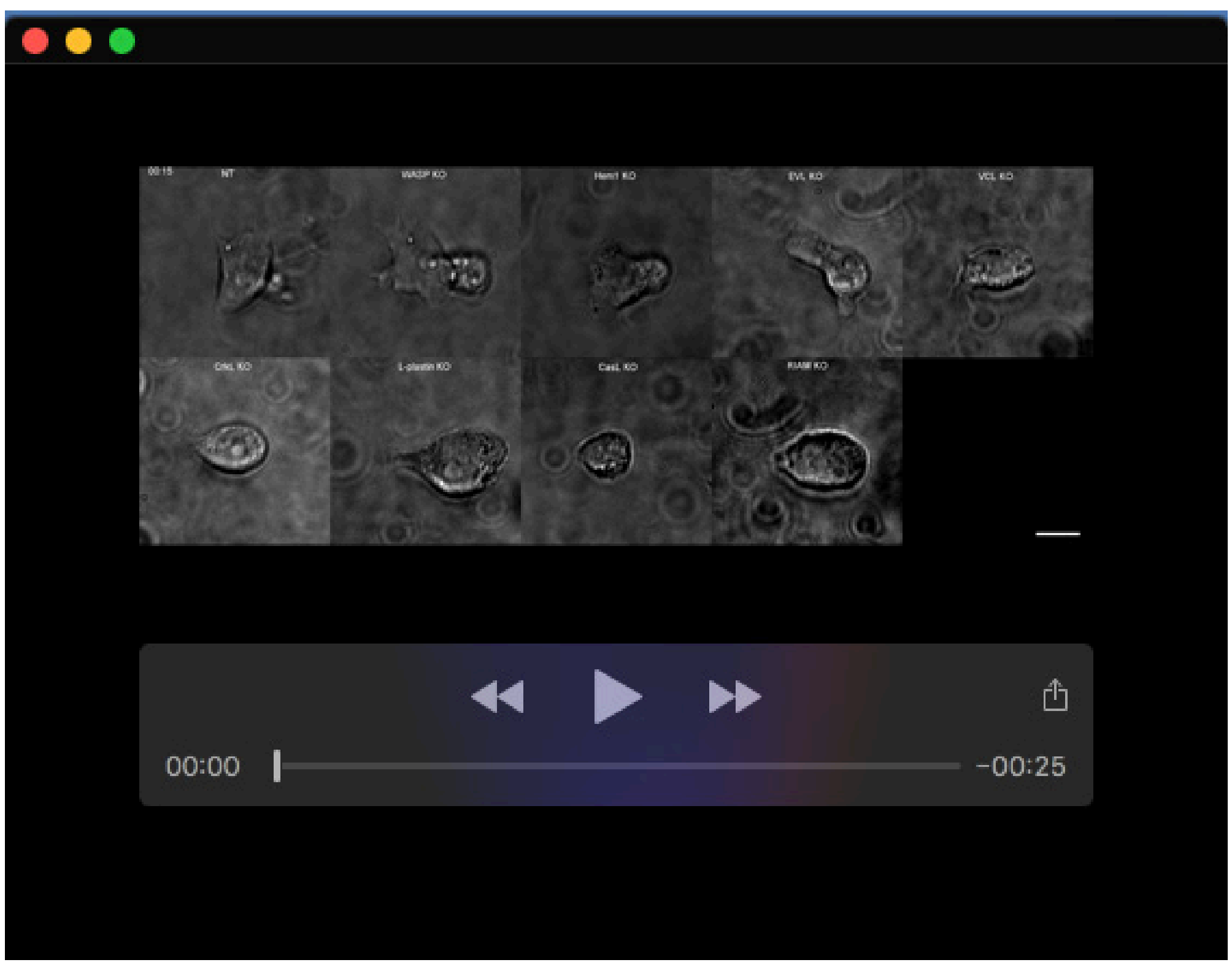

Movie 1. Representative DIC videos of KG-1a knockouts migrating under shear flow. Representative DIC videos of KG-1a NT, WASP KO, Hem1 KO, EVL KO, VCL KO, CrkL KO, L-plastin KO, CasL KO, and RIAM KO cells migrating on ICAM-1. Images were captured at 1s intervals for 1 min. 800 s^{-1} shear flow is from left to right, and the scale bar is 10 μm.

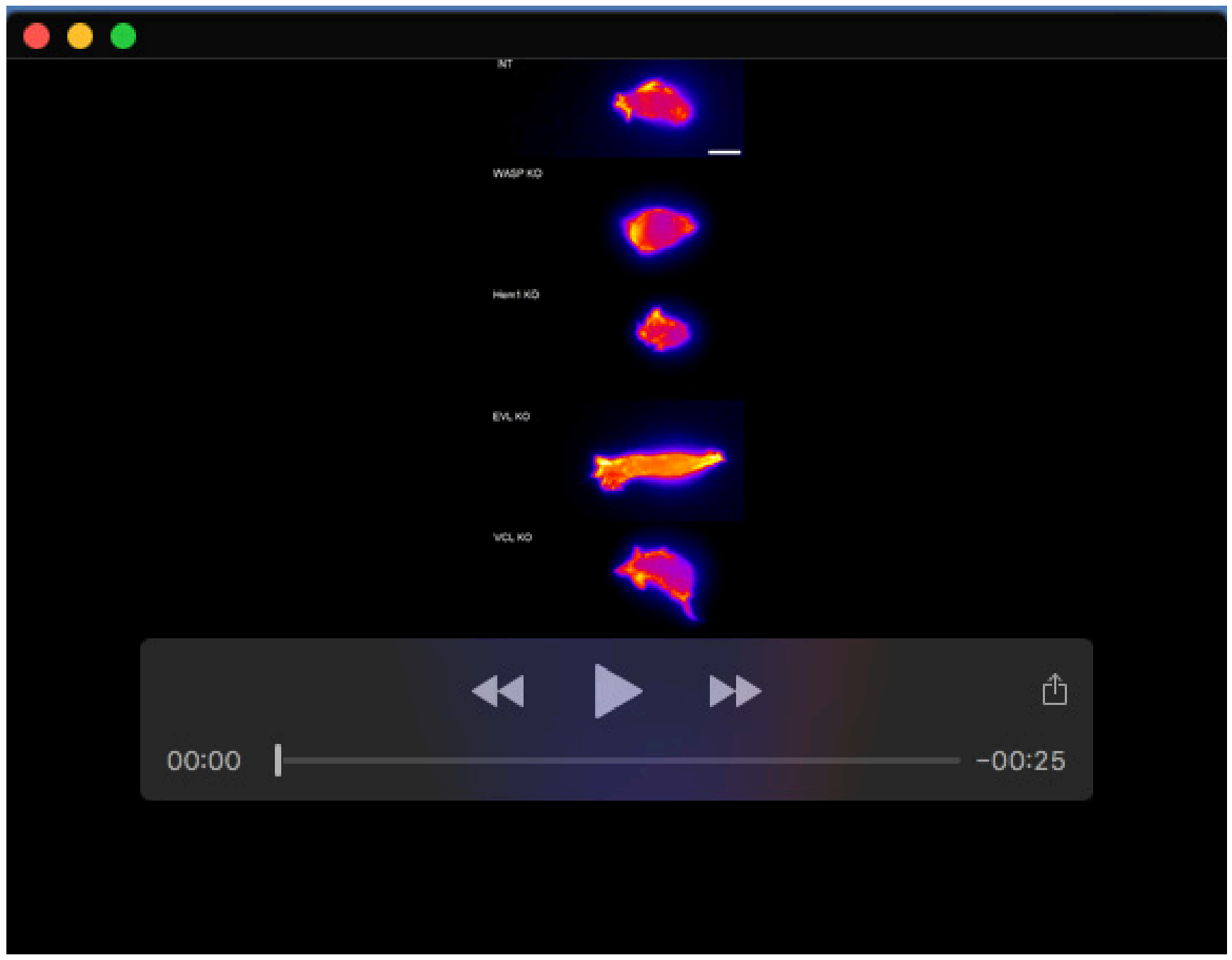

Movie 2. Representative Lifeact-GFP expressing KG-1a knockouts migrating upstream. Lifeact-GFP expressing KG-1a Non-targeting (NT), WASP KO, Hem1 KO, EVL KO, and Vinculin (VCL) KO cells migrating upstream on ICAM-1. Images were captured at 5s intervals for 3 min. 800 s^{-1} shear flow is from left to right, and the scale bar is 10 μm.

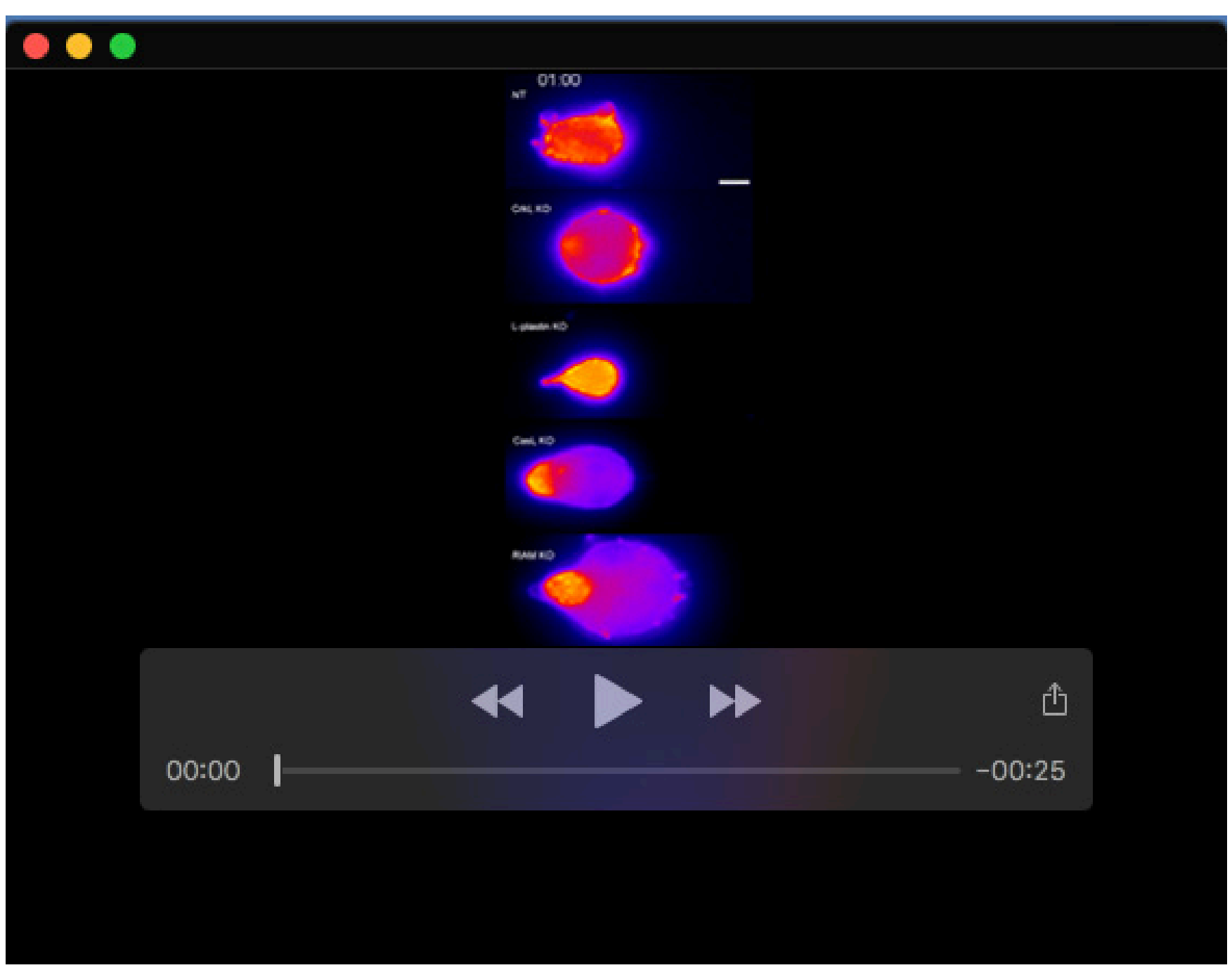

Movie 3. Representative Lifeact-GFP expressing KG-1a knockouts migrating downstream. Lifeact-GFP expressing KG-1a Non-targeting (NT), CrkL KO, L-plastin KO, CasL KO, and RIAM KO cells migrating downstream on ICAM-1. Images were captured at 5s intervals for 3 min. 800 s^{-1} shear flow is from left to right, and the scale bar is 10 μm.
